# Visualization of *in vivo* metabolic flows reveals accelerated utilization of glucose and lactate in penumbra of ischemic heart

**DOI:** 10.1038/srep32361

**Published:** 2016-09-01

**Authors:** Yuki Sugiura, Yoshinori Katsumata, Motoaki Sano, Kurara Honda, Mayumi Kajimura, Keiichi Fukuda, Makoto Suematsu

**Affiliations:** 1 Department of Biochemistry, Keio University School of Medicine, Tokyo, 160-8582, Japan; 2 Precursory Research for Embryonic Science and Technology (PRESTO), Japan Science and Technology Agency, Tokyo, 102-0076, Japan; 3 Department of Cardiology, Keio University School of Medicine, Tokyo, 160-8582, Japan; 4 Exploratory Research for Advanced Technology (ERATO), Japan Science and Technology, Suematsu Gas Biology Project, Tokyo, 102-0076, Japan

## Abstract

Acute ischemia produces dynamic changes in labile metabolites. To capture snapshots of such acute metabolic changes, we utilized focused microwave treatment to fix metabolic flow *in vivo* in hearts of mice 10 min after ligation of the left anterior descending artery. The left ventricle was subdivided into short-axis serial slices and the metabolites were analyzed by capillary electrophoresis mass spectrometry and matrix-assisted laser desorption/ionization imaging mass spectrometry. These techniques allowed us to determine the fate of exogenously administered ^13^C_6_-glucose and ^13^C_3_-lactate. The penumbra regions, which are adjacent to the ischemic core, exhibited the greatest adenine nucleotide energy charge and an adenosine overflow extending from the ischemic core, which can cause ischemic hyperemia. Imaging analysis of metabolic pathway flows revealed that the penumbra executes accelerated glucose oxidation, with remaining lactate utilization for tricarboxylic acid cycle for energy compensation, suggesting unexpected metabolic interplays of the penumbra with the ischemic core and normoxic regions.

Matrix-assisted laser desorption/ionization (MALDI)-imaging mass spectrometry (IMS); also referred to as mass spectrometry imaging, has been developed as a sensitive technique for *in vivo* imaging of individual biomolecules. It was initially developed for proteome analyses^1^,^2^,^3^, and then subsequently used to study the lipidome^4^,^5^,^6^,^7^. Our laboratory has focused on two-dimensional analysis of central metabolism with high spatial resolution using MALDI-IMS. The capability of MALDI-IMS to show the distribution of primary metabolites was first demonstrated on rat brain sections using a 9-aminoacridine matrix^8^. We developed a quantitative IMS technique by combining IMS and capillary electrophoresis (CE)-electrospray ionization (ESI)-mass spectrometry (MS), and showed spatiotemporal changes in adenylates (ATP, ADP, AMP) and NADH on brain sections of mice in response to acute cerebral ischemia^9^.

To accurately assess highly labile metabolites, rapid inactivation of intracellular metabolism is critical. Ischemic changes in energy metabolites following circulatory arrest are more rapid in the heart than in other non-pulsatile organs. Extraction of the heart following cervical dislocation takes at least several seconds, rendering the oxidative phosphorylation-dependent heart tissue hypoxic; such circumstances significantly alters metabolites^10^. Hori S. *et al*. developed an automatic device that makes cross-sectional slices from canine beating hearts *in situ* at a predetermined phase of the cardiac cycle and fixes metabolites by freeze-clamping, all within 120 ms^11^. The drawback of this method is that only a single slice may be obtained from a dog. Evaluation of sequential heart sections from a smaller animal, such as a mouse, has been impossible. To obtain a two-dimensional display of highly labile metabolites in heart tissues *in vivo*, the metabolic activity must be rapidly terminated *in vivo* to minimize ischemic artifacts.

The focused microwave irradiation (FMW) technique has been developed to eliminate biochemical artifacts resulting from animal tissue extraction^12^. The primary mechanism of FMW is the rapid heating of tissues to about 80 °C, which inactivates many enzymatic reactions while retaining tissue integrity^13^. It is useful in the fixation of energy metabolites that would otherwise fluctuate widely following animal sacrifice by decapitation^14^.

In this study, we focused on acute phases of ischemia where changes in metabolism are more dynamic than at later phases, including the post-infarct stages. Since many metabolites that change during the early phase of ischemia (e.g. adenylates, lactate, succinate, glutamate, malate, and NADH) are highly labile, it was necessary to develop a new heart preparation method for MALDI-IMS. For example, ATP is degraded within seconds, whereas creatine and lysophospholipids^7^, are less labile in this timescale^15^. Combining the FMW fixation method with MALDI-IMS and CE-MS made it possible to capture metabolite levels, and to track the *in vivo* fate of exogenously administered glucose and lactate that were labeled with ^13^C-isotope, with high spatial resolution in and around the ischemic myocardium.

## Results

### FMW is essential for deciphering the metabolic dynamics of an ischemic heart

**Figure 1:**
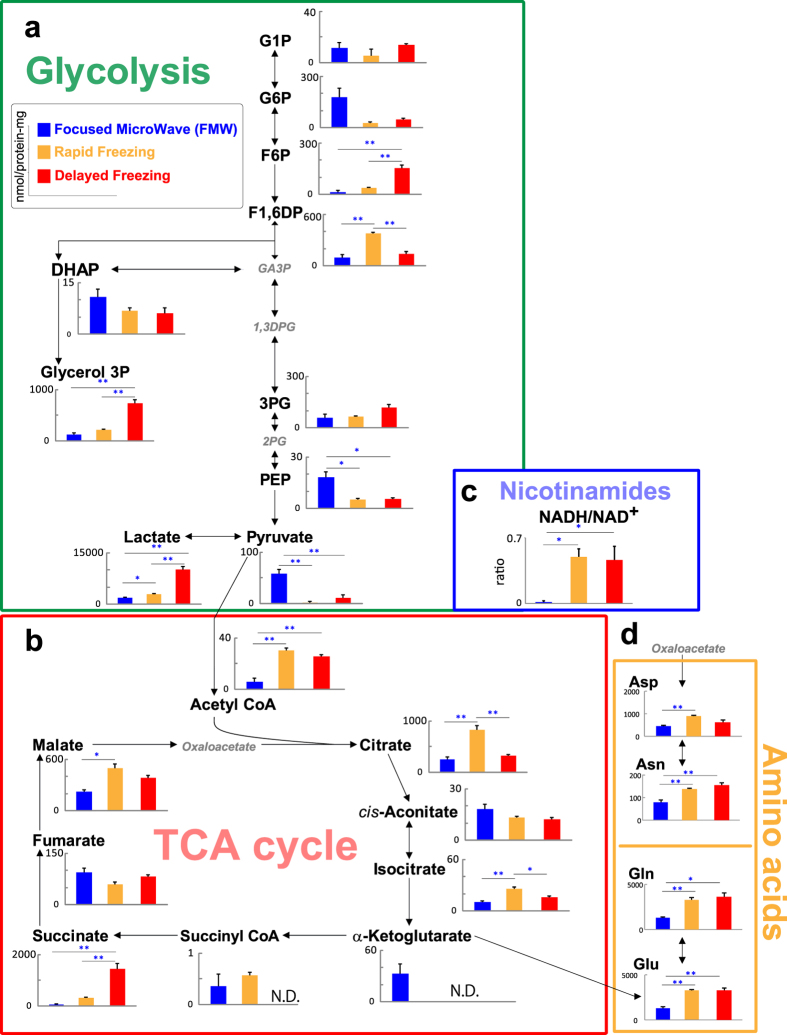
Focused microwave irradiation is the most suitable tissue fixation method. Hearts were prepared by three different methods: focused microwave irradiation (FMW), rapid freezing method, in which hearts were isolated immediately after thoracotomy and then quick-frozen in liquid N_2,_ and by delayed freezing, whereby hearts were isolated 10 min after cervical dislocation. The time required for inactivation of postmortem enzymatic activity was shortest in FMW (0.96 s), followed by rapid freezing (approx. 20 s after thoracotomy) and delayed freezing (10 min after cervical dislocation). (**a**–**c**) Amounts of metabolites in the glycolytic pathway(**a**), tricarboxylic acid (TCA) cycle (**b**), values for NADH/NAD^+^ ratio (**c**) and amino acids (**d**) were determined by CE-MS. *P < 0.05, **P < 0.01 (n = 6).

We first examined whether FMW is superior to traditional sample preparation in terms of preventing metabolic artifacts (see Supplementary Fig. S1 for details of the heart-focused FMW method). We compared a broad range of metabolites in glycolysis and the tricarboxylic acid (TCA) cycle, and the NADH/NAD^+^ redox state in heart homogenates obtained by three different methods: FMW, rapid freezing (traditional sample preparation) and delayed-freezing methods. Cardiac metabolites were quantified by CE-MS (Fig. 1).

At 10 min *post mortem*, lactate production (Fig. 1a) and the NADH/NAD^+^ ratio in the heart increased markedly (red bars, Fig. 1c). Succinate accumulated as an end product of anaplerotic and reverse reactions of the TCA cycle (Fig. 1b)^16^,^17^. FMW was superior to traditional methods of sample preparation at preventing *post-mortem* ischemic changes (blue bars vs. yellow bars, Fig. 1a–c). Traditional sample preparation by rapid freezing produced a considerable increase in lactate production, and an accumulation of TCA intermediates (acetyl-CoA, citrate, isocitrate, succinate, malate) compared to FMW (Fig. 1b). Of particular importance, the NADH/NAD^+^ ratio in the hearts obtained by the rapid-freezing method was elevated to the same level as in hearts obtained by the delayed-freezing method. In contrast, the NADH/NAD^+^ ratio remained markedly lower (P < 0.01) in the hearts prepared by FMW (blue bar, Fig. 1c) than in those obtained by the rapid-freezing method (yellow bar). Together, these findings indicate that FMW is essential during the sacrifice to minimize *post-mortem* ischemic changes.

### Two-dimensional mapping of central metabolites on multiple sequential sections of a heart subjected to acute regional ischemia

To examine the region-specific changes in metabolites at the early phase of ischemia, hearts were fixed by FMW after ligating the left anterior descending artery (LAD). The left ventricle was divided into short-axis blocks that were analyzed by both CE-MS and MALDI-IMS (see Supplementary Fig. S2). For intergroup comparison, MALDI-IMS of metabolites in tissue sections was normalized by CE-MS-based quantitative data obtained from adjacent tissue blocks (i.e., semi-quantitative MALDI-IMS^9^). The outlines of the right and left ventricular cavities, including two papillary muscles, were well preserved in tissue sections obtained from FMW-fixed hearts, whereas the ventricular cavities were crushed in tissue sections obtained from non-FMW-fixed hearts (see Supplementary Fig. S3).

**Figure 2:**
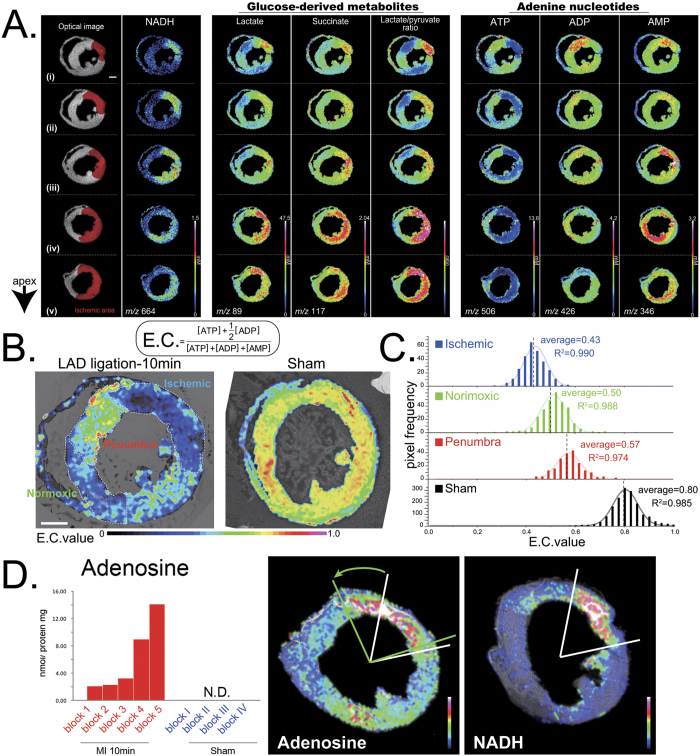
Quantitative imaging of cardiac metabolites in ischemic hearts. (**a**) Representative images of NADH, lactate, succinate, lactate/pyruvate ratio, ATP, ADP, AMP are shown. The red region in the optimal image indicates the area with increased NADH as a metabolic indicator for the ischemic region. We repeated the experiments three times with reproducible results (see Supplementary Fig. S10). (**b**) Mapping of energy charge (EC) values based on MALDI-IMS, normalized using capillary electrophoresis (CE)-MS-based quantitative analysis. Representative images of sham-operated (sham) or 10 min post–myocardial infarction (MI) mice are shown. Experiments were repeated three times with reproducible results (see Supplementary Fig. S10). (**c**) The EC map was subdivided into three sectors: a region of ischemia (ischemic), a region adjacent to ischemia (penumbra), and a region distant to ischemia (normoxic). Values for EC in these sectors are shown as histograms. Sham, a heart obtained from a sham-operated mouse. (**d**) Elevated production of adenosine was detected in correlation with ischemic severity (left), which overflowed into the penumbra region from the ischemic core (arrow in right panel).

At 10 min after LAD ligation, molecular images of lactate, succinate, lactate/pyruvate ratio, NADH and ATP showed a sharp line of demarcation between ischemic and non-ischemic myocardium (Fig. 2a). We used the increment of NADH, the most sensitive redox marker, as a metabolic indicator of the ischemic region (Fig. 2a, optical image). The region adjacent to ischemia (penumbra region) had higher ATP and ADP levels, and a lower lactate/pyruvate ratio than the region more distant to ischemia (normoxic region). An energy charge (EC) map of LAD-ligated hearts demonstrated three metabolically different ischemic, penumbra and normoxic regions (Fig. 2b). The EC values in these regions are presented in a histogram (Fig. 2c). The EC in the non-ischemic myocardium was lower than in the normal myocardium of a sham-operated mouse and lowest in the ischemic myocardium core. Consistent with quantitative images of ATP, the penumbra region exhibited a higher EC than the normoxic region. We also found that adenosine, a strong vasodilator, was produced not only in the ischemic core but was diffused into the penumbra region (Fig. 2d), resulting in intense ischemic hyperemia in this region.

### Tracing glucose metabolic pathways in a designated region of the heart

**Figure 3:**
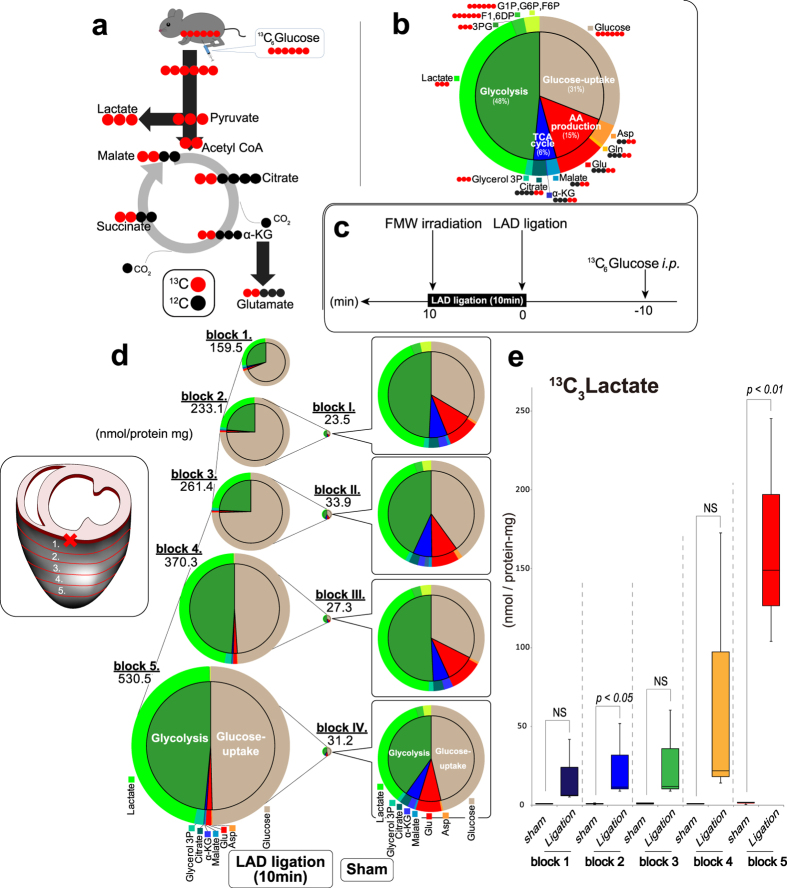
Pathway tracing analysis using ^13^C_6_-glucose in ischemic hearts. (**a**) Schematic representation of ^13^C_6_-glucose utilizing pathway. (**b**) Cardiac metabolites were fixed by focused microwave irradiation (FMW) 25 min after injecting ^13^C_6_-glucose. The inner and outer pies indicate the fractions of ^13^C-labeled metabolites that belong to the specified metabolic pathways and those of each metabolite species. Grey indicates glucose uptake, red amino acid (AA) production (Asp, Gln, Glu), blue tricarboxylic acid (TCA) cycle (citrate, 2-DG, malate) and green glycolysis (G1P, G6P, F6P, F1, 6DP, 3PG, lactate, glycerol 3P). Note that a large fraction of glucose is diverted to the syntheses of glutamate and lactate. Experiments were repeated at least three times with consistent results. Representative pie charts are shown. (**c**) Experimental protocol for generating the left anterior descending artery (LAD) ligation model. Mice were subjected to LAD ligation or a sham operation at 10 min after injecting ^13^C_6_-glucose (1 mg/g body weight in saline, i.p.). After 10 min, myocardial metabolites were snap-fixed *in vivo* by FMW. (**d**) Capillary electrophoresis–mass spectrometry (CE-MS) quantification of ^13^C-containing metabolites in the respective blocks are represented as pie charts. Experiments were repeated at least four times with consistent results. Representative pie charts are shown (see Supplementary Fig. S11). (**e**) Absolute quantification of ^13^C_3_ lactate in the respective blocks of LAD-ligated or sham-operated mice (n = 4, for each). Abbreviations: G1P, glucose-1-phosphate; G6P, glucose-6-phosphate; F6P, fructose-6-phosphate; F1,6DP, fructose-1,6-disphosphate; 3PG, 3-phosphoglycerate; Glycerol 3P, glycerol-3-phosphate; α-KG, α-ketoglutarate; Gln, glutamine; Glu, glutamate; Asn, asparagine; Asp, aspartic acid.

To examine regional differences in the metabolic fate of glucose at the early phase of ischemia, we measured ^13^C-labelled metabolites in heart blocks from different locations between the heart base and apex by CE-MS (Fig. 3a,c–e). First, we characterized the metabolic fates of glucose in normal beating hearts (Fig. 3a,b). Under steady-state conditions, a significant proportion of TCA cycle intermediates was diverted to ^13^C_2_-glutamate, indicating a substantial portion of α-ketoglutarate was converted to glutamate. Therefore, we used carbon flow from ^13^C_6_-glucose into ^13^C_2_-glutamate as a marker of TCA cycle entry in the following experiments.

We then compared the metabolic fate of glucose in LAD-ligated or sham-operated hearts (Fig. 3c). At 10 min after LAD ligation, glucose consumption at the apex of LAD-ligated hearts was 15-20-fold higher than in sham-operated hearts (Fig. 3d). Lactate production was markedly increased in LAD-ligated hearts compared to sham-operated hearts (Fig. 3d,e) and was greatest at the cardiac apex and lowest at the cardiac base. The proportion of intermediates in the TCA cycle and their derivatives was lower in LAD-ligated hearts than in sham-operated hearts.

**Figure 4:**
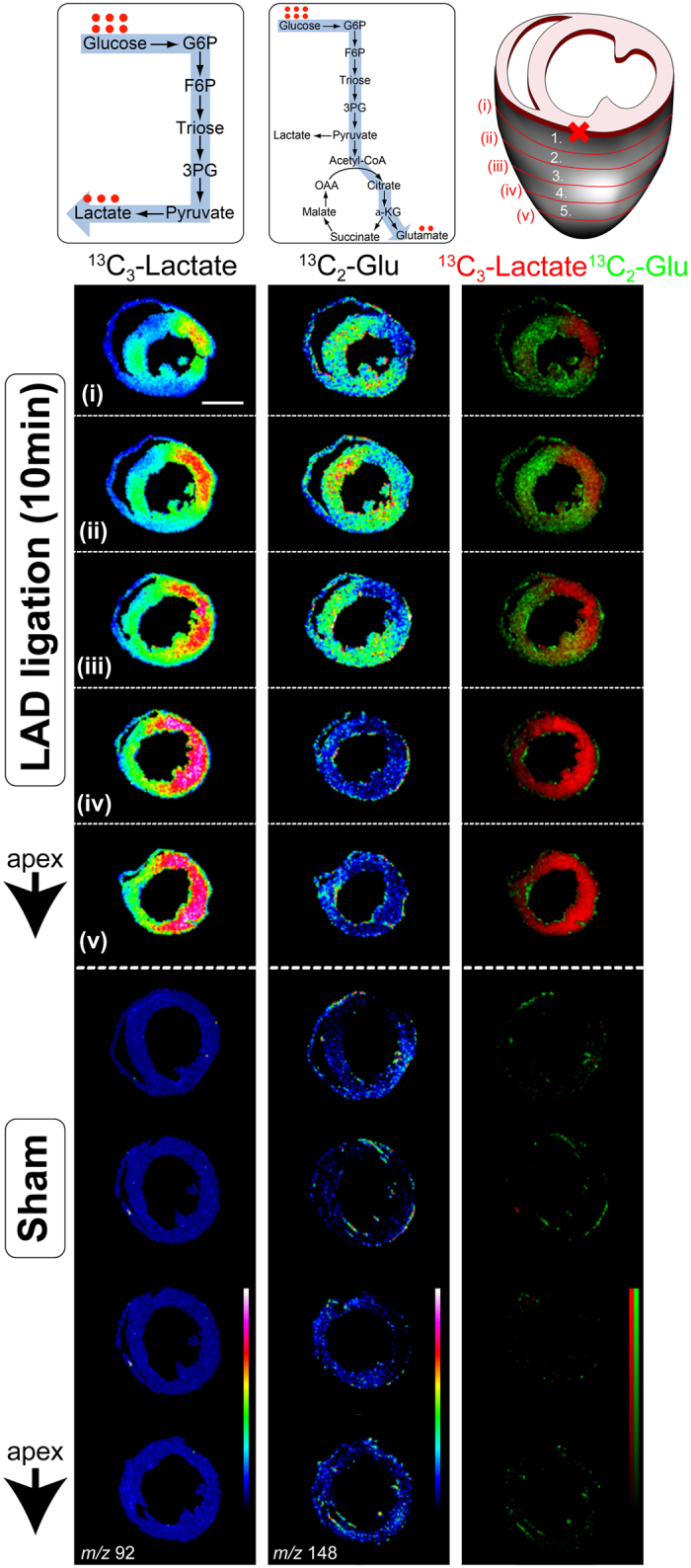
Distinct glucose metabolic pathway utilization in ischemic hearts. Two-dimensional maps of ^13^C-labelled metabolites were reconstructed from MALDI-IMS combined with 9-aminoacridine as a matrix, and shown as an overlay on optical heart section images. Images were normalized with total ion current (TIC). The right lane is the merge of ^13^C_3_-lactate and ^13^C_2_-glutamate. Experiments were repeated at least four times with reproducible results. Representative images are shown. Abbreviations: Glu, glutamate.

Next, we sought to visualize a variety of ^13^C-labelled intermediates in glycolysis and the TCA cycle in heart sections 10 min after LAD ligation by MALDI-IMS using either 9-aminoacridine or N-(1-naphthyl) ethylenediamine dihydrochloride (NEDC) as a matrix (Fig. 4 and Supplementary Fig. S4). NEDC allowed us to visualize the distribution of organic acids in heart sections, such as ^13^C_2_-succinate and ^13^C_2_-citrate^18^, that are typically difficult to detect by 9-aminoacridine utilizing MALDI-IMS. The ischemic area was clearly demarcated by an accumulation of ^13^C_2_-lactate and ^13^C_2_-succinate, and by the reduction of ^13^C_2_-citrate and ^13^C_2_-glutamate. ^13^C_2_-lactate was markedly elevated in the ischemic region (Fig. 4 and Supplementary Fig. S4). However, the elevation of ^13^C_2_-lactate was also observed in the non-ischemic region of LAD-ligated mice compared to the normally functioning myocardium. In contrast, ^13^C_2_-glutamate was elevated only in the non-ischemic region of LAD-ligated mice (Fig. 4). These findings indicate that anaerobic glycolysis is activated in the ischemic region, whereas both glycolysis (lactate production) and the TCA cycle are activated in the non-ischemic myocardium. The penumbra region contained more ^13^C_2_-glutamate, suggesting that the entry of glucose metabolites into the TCA cycle is enhanced in this region compared to the distant normoxic region (Fig. 4). These results were consistent with the penumbra region having greater EC values than the distant normoxic region.

### Tracing lactate metabolic pathways in a designated region of the heart

**Figure 5:**
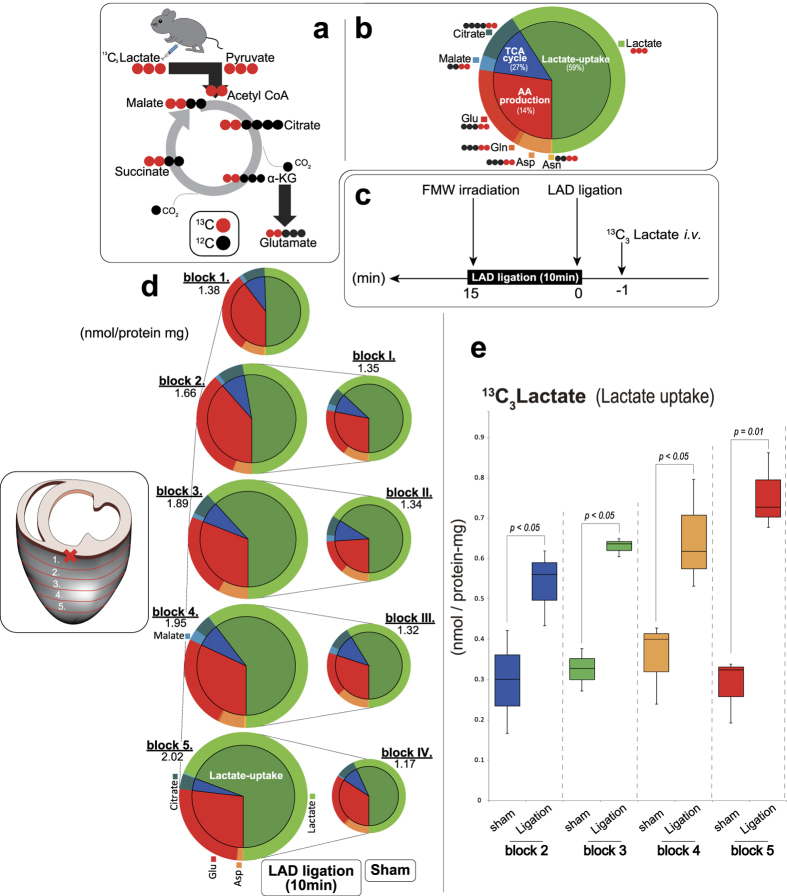
Pathway tracing analysis using ^13^C_3_-lactate in the heart following LAD ligation. (**a**) Schematic representation of ^13^C_3_-lactate utilizing pathway. (**b**) Heart metabolites were fixed by focused microwave irradiation (FMW) 1 min after injecting ^13^C_3_-lactate. The inner and outer pies indicate the fractions of ^13^C-labeled metabolites that belong to the specified metabolic pathways and those of each metabolite species. The green area indicates lactate uptake (lactate), red amino acid (AA) production (Asn, Asp, Gln, Glu), and blue tricarboxylic acid (TCA) cycle activity (citrate, malate). Experiments were repeated at least four times with consistent results. Representative pie charts are shown. (**c**) Experimental protocol for generation of left anterior descending coronary artery (LAD) ligation model. 1 min after injecting ^13^C_3_-lactate (1 mg/g body weight in saline, i.v.), mice were subjected to LAD ligation or a sham operation. 10 min later, myocardial metabolites were snap-fixed *in vivo* by FMW. (**d**) Capillary electrophoresis–mass spectrometry (CE-MS) quantification of ^13^C-containing metabolites in the respective blocks is represented by pie charts. Experiments were repeated at least four times with consistent results. Representative pie charts are shown. The inner and outer pies indicate the proportion of ^13^C-labeled metabolites that belong to the specified metabolic pathways and those of each metabolite species. (**e**) Absolute quantification of ^13^C_3_-lactate uptake among the respective blocks of four LAD-ligated or sham-operated mice (n = 4, for each). Abbreviations: Glycerol 3P, glycerol-3-phosphate; Gln, glutamine; Glu, glutamate; Asn, asparagine; Asp, aspartic acid.

The heart releases lactate generated through glycolysis while simultaneously taking up exogenous lactate and oxidizing it in mitochondria under both stable and ischemic conditions. To examine the regional differences in the metabolic fate of lactate during the early phase of ischemia, we measured ^13^C-labelled metabolites in heart blocks from different locations between the cardiac base and apex by CE-MS (Fig. 5a,c–e). We first characterized the metabolic fate of lactate in normal beating hearts (Fig. 5a,b). Under steady-state conditions, exogenous ^13^C_3_-lactate became a source of energy by entering the TCA cycle. We were unable to detect any ^13^C-labeled intermediate metabolites of glycolysis or those of the pentose phosphate pathway. We then examined the metabolic fate of lactate in LAD-ligated or sham-operated hearts (Fig. 5d). Lactate consumption in LAD-ligated hearts was two to three times higher than in sham-operated hearts (Fig. 5d,e). The increased lactate uptake in LAD-ligated mice was modest when compared to the increase in glucose uptake (Figs 3d and 5d). Lactate uptake was greatest at the cardiac apex and lowest at the base, though the difference was modest.

**Figure 6:**
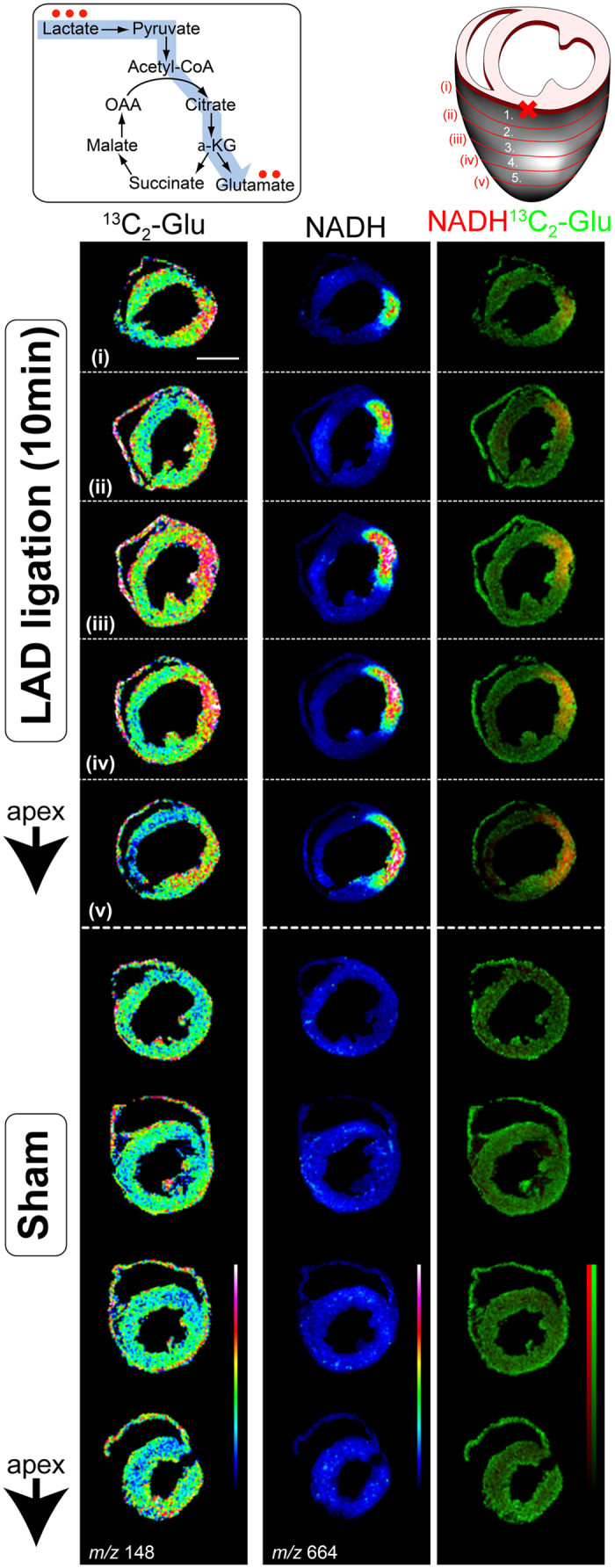
Conversion of exogenous lactate into glutamate in ischemic core. Two-dimensional maps were reconstructed from matrix-assisted laser desorption/ionization imaging mass spectrometry (MALDI-IMS) imaging of metabolites, and shown as an overlay in optical heart section images. Images were normalized with total ion current (TIC). Representative images are shown. NADH imaging was used to determine the region of ischemic myocardium. The right lane is the merge of ^13^C_2_-glutamate and NADH. Experiments were repeated at least four times with reproducible results. Abbreviations: Glu, glutamate.

Visualizing the fate of ^13^C-labelled lactate on heart sections 10 min after LAD ligation by MALDI-IMS demonstrated unexpectedly high levels of ^13^C_2_-labelled glutamate in the ischemic region as compared to that in the non-ischemic region (Fig. 6). In the non-ischemic region, this conversion of lactate into glutamate was comparable to that observed in sham-operated hearts. There was no difference in the intensity of ^13^C-labelled glutamate between the penumbra and normoxic regions. These results indicate that lactate production and lactate utilization are differentially regulated during the acute phase of regional myocardial ischemia.

## Discussion

MALDI-IMS has been applied to map the spatial distributions of non-labile metabolites using fixation methods that are prone to autolysis. In this study, our intention was to capture snapshots of metabolic derangements during an acute phase of ischemia when metabolic alterations are more dynamic than in later phases. We developed a methodology to map regional differences in metabolic responses 10 min after LAD ligation; before death and necrosis of myocardial tissue occur^19^. We demonstrated that FMW is superior to traditional methods of heart preparation for preserving labile metabolites and thus, maintaining their relative *in vivo* abundance. Optimization of the FMW cardiac fixation conditions enabled us to reveal region-specific derangements of energy metabolites in the ischemic- and peri-core of compromised heart tissues. Furthermore, metabolite maps reconstructed using CE-MS-based quantitative data allowed intergroup comparison. Application of ^13^C-labelling to IMS also enabled us to trace metabolic pathways in a designated region of the heart. Our study visualized for the first time various ^13^C-labelled intermediates in glycolysis and the TCA cycle. Using NEDC as a matrix reagent allowed us to visualize ^13^C_2_-succinate and ^13^C_2_-citrate in heart sections at concentrations that are undetectable by 9-aminoacridine-assisted MALDI-IMS.

**Figure 7:**
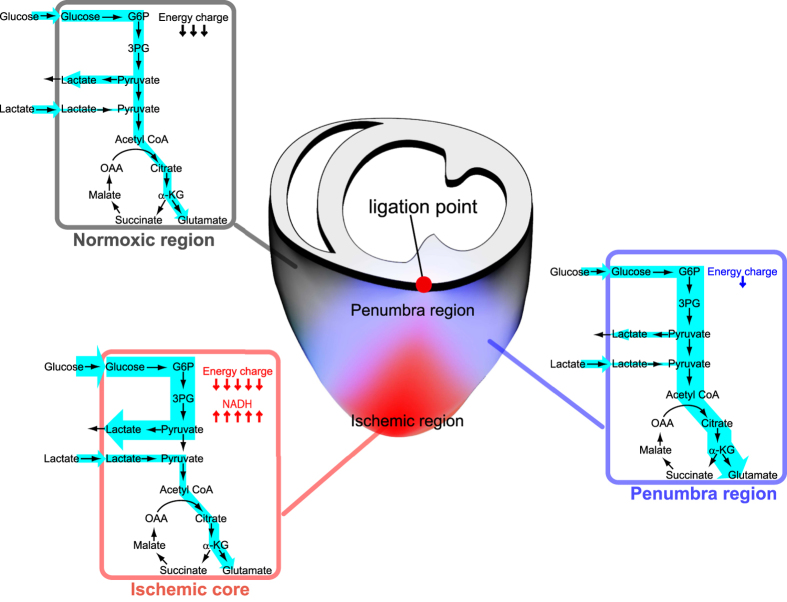
A diagram showing metabolism in the ischemic, penumbra and distant normoxic regions. Heterogeneity in carbohydrate metabolism in and around ischemic myocardium in the early phase of left anterior descending coronary artery (LAD) ligation. Ischemic region: • A marked reduction in energy charge due to impaired energy production in mitochondria due to lack of oxygen (NADH, succinate accumulation). • A large increase in glucose uptake, an accelerated rate of glycolysis, and lactate production. • Lactate uptake is maintained and its catabolism using the Krebs cycle increases. Non-ischemic region (including penumbra and normoxic region): • A lower energy charge than a normal myocardium from a sham-operated mouse due to high energy expenditure to compensate functionally for the loss of working myocardium. • An increase in the uptake of glucose and lactate. • Activation of glycolysis and entry of glycolytically derived pyruvate into the Krebs cycle. Penumbra region: • Higher energy charge and a lower lactate/pyruvate ratio than regions more distant. • The preferential entry of glycolytically derived pyruvate into the Krebs cycle.

Applying these technologies, we were able to define three metabolically distinct regions in the acute phase of myocardial ischemia 10 min after LAD ligation: the ischemic core, the non-ischemic region adjacent to the core (penumbra region) and the non-ischemic region more distant to ischemia (normoxic region). Imaging analysis of metabolic pathway flows revealed that the penumbra executes unexpected metabolic interplays with ischemic and normoxic regions (Fig. 7). The ischemic region exhibited accelerated anaerobic glycolysis and reduced glucose oxidation as expected. The normoxic region was energetically compromised despite the activation of glycolysis and the entry of glucose metabolites into the TCA cycle as compared to sham controls. We speculate that the normoxic region has to consume more energy to compensate functionally for the loss of a working myocardium^20^,^21^. Of interest was the unique metabolism of the penumbra region, which exhibited higher EC and a lower lactate/pyruvate ratio than the distant normoxic region as a result of the preferential entry of glucose metabolites into TCA cycle. Rees and Redding reported that following coronary arterial occlusion, blood flow to muscle immediately surrounding the ischemic muscle was greater than the blood flow to muscle more distant to the ischemic region^22^. This disproportionate increase in blood flow could be explained by localized vasodilation due to greater oxygen consumption near the infarct, the diffusion of vasodilator metabolites (e.g. adenosine) from the infarcted regions, a localized reduction in tissue pressure or a reflex.

Our approach detected a surprising and specific increase in ^13^C_3_-glutamate at the ischemic core that appears to be derived from blood-borne ^13^C_3_-lactate (as summarized in Fig. 6). Without molecular oxygen, cytochrome *c* oxidase in the electron transport chain cannot accept electrons from NADH. Considering that the heart apex exhibited a marked increase in NADH, it is most likely that O_2_ concentrations were below the K_m_ value for O_2_ in cytochrome *c* oxidase (1 μM, pO_2_ < 1 mmHg). At the apex, the region most severely challenged by ischemia, oxidative phosphorylation would therefore be very limited. Lactate cannot be oxidized to produce ATP. Mechanisms that result in the apex exhibiting substantial amounts of ^13^C_3_-lactate derived from ^13^C_3_-glutamate are unknown and deserve further investigation.

Metabolic change during acute heart ischemia has been extensively studied. However, our study is one of the first to conduct a non-targeted approach to unraveling the control points in metabolic pathways during acute ischemia by systematic metabolic profiling. Currently, the number of metabolites that can be visualized by IMS is smaller than that measurable by CE-MS (see Supplementary Table S1). This is due to the nature of sample complexity in tissue sections, which consist of an extremely heterogeneous mixture of biomolecules. Moreover, in IMS, the sample cleanup procedure is limited, whereas in quantitative MS analysis, analyte molecules are extracted and separated from crude samples by chromatography or electrophoresis. When such a crude sample is subjected to IMS, numerous molecular species compete for ionization, and molecules that are easily ionized preferentially reach the detector. To overcome this issue, the optimization of sample conditions plays a critical role in efficient ionization of analyte molecules in the crude mixture. We recently established an on-tissue derivatization method, in which metabolites with low ionization efficiency are chemically derivatized so that they are detected with high sensitivity^23^. Taken together, we expect that continuous improvements in the experimental protocol, as well as in MS instrumentation, will further expand the potential of this emerging technique.

The search to understand the complex molecular mechanisms underlying heart disease has constantly driven researchers to develop new technologies. Various MS analyses have undoubtedly had a profound impact in many research laboratories, and will continue to do so in the future. Heart-focused microwave irradiation will be imperative for sample preparation in these techniques to preserve *in vivo* levels of labile metabolites while maintaining structural integrity. Our novel strategy should lead to better understanding of cardiac metabolism.

In conclusion, we have provided a protocol for FMW sacrifice, which enabled us to maintain *in vivo* concentrations and metabolite localization to image and quantify various metabolites using MS. We established MS-based technologies for quantifying and imaging ^13^C-labeled metabolites derived from ^13^C-labeled energy substrates, allowing us to determine predominant downstream metabolic pathways in *in vivo* tissues.

## Methods

### Chemicals

^13^C_6_-glucose and ^13^C_3_-lactate were obtained from ISOTEC (Sigma-Aldrich). N-(1-Naphthyl) ethylenediamine dihydrochloride and 9-aminoacridine were obtained from Merck Millipore. All other chemicals were obtained from either Sigma-Aldrich or Wako. All chemicals were HPLC reagent grade.

### Mouse model of myocardial ischemia

All animal procedures were conducted in accordance with the Animal Experimentation Guidelines of Keio University School of Medicine and were approved by the Laboratory Animal Care and Use Committee of Keio University [Permission number; 11050-(0), 12094-(1)]. Eight-week-old male C57BL/6J mice (22–26 g; Clea Japan, Tokyo, Japan) were used. They were fed with laboratory chow and allowed free access to water.

Myocardial ischemia was induced by LAD ligation as described previously^24^. For more detail, see the supplementary information.

### Administration of ^**13**^C-labelled metabolites

For experiments tracing the metabolic fate of glucose, mice received ^13^C_6_-glucose (1 mg/g body weight, in saline) by intraperitoneal administration 10 min before LAD ligation, because at this time point, blood glucose concentration is high enough to trace metabolic pathways of *in vivo* organs by MS^15^. ^13^C_3_-lactate (27 μg/g body weight, in saline) was administered by retro-orbital injection 1 min before LAD ligation^25^. We have confirmed the elevation of blood lactate concentration to 7–8 mM at this time point, which is within the physiological range (Supplementary Fig. S5)^26^.

### Fixation of heart metabolites by FMW

We used a laboratory microwave instrument (MMW-05 Muromachi Kikai, Tokyo) designed for the euthanasia of laboratory mice and rats (see Supplementary Fig. S1). This instrument differs from kitchen units, particularly in maximal power output (5 kW) and in having a tightly focused microwave beam. All units direct their microwave energy to a specific anatomical location on the animal. Mice were anesthetized with isoflurane and placed into a transparent water-jacket holder (Muromachi-Kikai, MH28-HZ). The cone-parts of the holder were filled with ~1 mL of water to help elevate the temperature of the heart as uniformly as possible using microwave energy. Care was taken not to introduce air bubbles. This was then inserted into the instrument in a position such that microwave irradiation was targeted on both brain and heart. For reliable fixation, maintaining the animal in the correct position is very important; i.e., the mouse should be straight with its nose touching the top of the cone. The holder is set at the position shown in Supplementary Fig. S1, panel-D; the back end of the holder was set at 43 mm from the entrance of the insertion slot. We found this condition optimal for B6/J mice (8-week old males). Microwave irradiation at 5 kW for 0.96 s elevated the temperature of the heart to above 80 °C, which is sufficient to inactivate metabolic enzymes such as acetylcholine esterase^13^.

To evaluate the effectiveness of the FMW fixation method, we compared it to two other procedures: i) rapid-freezing, in which hearts were isolated immediately after thoracotomy then frozen in liquid N_2_ (total procedure takes ~20 s); ii) delayed freezing, in which hearts were isolated 10 min after cervical dislocation to allow postmortem degradation.

### Preparation of tissue sections for metabolome and MALDI-imaging analyses

After FMW, hearts were dissected with a surgical knife at room temperature, embedded into a super cryo-embedding medium (SCEM, Section Lab Co. Ltd, Hiroshima, Japan), frozen in liquid N_2_, and stored at −80 °C. We prepared five sets of short-axis (transverse) tissue sections where each set consisted of a thick 450 μm “block” for quantification of metabolites and an adjacent 8 μm thin “section” for MALDI imaging analyses (see Supplementary Fig. S6). The apical two-thirds of the left ventricle in sham-operated hearts was subdivided into four short-axis blocks.

The thin sections were cut with a cryomicrotome (CM3050, Leica Microsystems) and thaw-mounted on an indium thin oxide-coated glass slide (BrukerDaltonics, Germany) at −16 °C. Heart tissues subjected to FMW tended to be more fragile than those treated by other methods, often making tissue sectioning challenging. However, embedding the tissue with SCEM medium, which did not interfere with the ionization efficiency of metabolites, helped achieve successful sectioning.

### Capillary electrophoresis-electrospray ionization (CE-ESI)-MS

Quantitative metabolome analysis was performed using CE-MS^27^. Briefly, to extract metabolites from the tissue, the frozen tissue block embedded in SCEM medium together with internal control compounds (see below) was homogenized in ice-cold methanol (500 μL) using a manual homogenizer (Finger Masher (AM79330); Sarstedt, Tokyo, Japan), followed by the addition of an equal volume of chloroform and 0.4 times the volume of ultrapure water (LC/MS grade; Wako). The suspension was then centrifuged at 15,000 *g* for 15 min at 4 °C. After centrifugation, the aqueous phase was ultrafiltered using an ultrafiltration tube (Ultrafree-MC, UFC3 LCC NB; Human Metabolome Technologies, Tsuruoka, Japan). The filtrate was concentrated with a vacuum concentrator (SpeedVac; Thermo, Yokohama, Japan); this condensation process helps quantitate trace levels of metabolites. The concentrated filtrate was dissolved in 50 μL of ultrapure water and used for CE-MS.

All CE-MS experiments were performed using an Agilent CE System equipped with an air pressure pump, an Agilent 6520 Accurate Q-Tof mass spectrometer, an Agilent 1200 series isocratic high-performance LC pump, 7100 CE-system, a G1603A Agilent CE-MS adapter kit, and a G1607A Agilent CE-MS sprayer kit (Agilent Technologies). For more detail, see the supplementary information.

### Liquid chromatography-tandem mass spectrometry

The amount of non-labeled and ^13^C_6_-glucose in the heart was quantified using liquid chromatography-tandem mass spectrometry (LC-MS/MS). For more detail, see the supplementary information.

### Matrix coating and MALDI-IMS acquisition

Prior to matrix coating, the tissue slices were placed in desiccant for 10 min and allowed to equilibrate to room temperature. We used 9-aminoacridine as a matrix (10 mg/mL, dissolved in 80% ethanol) and manually spray-coated tissue sections with the solution using an artistic airbrush (Procon Boy FWA Platinum 0.2-mm caliber airbrush, Mr. Hobby, Tokyo, Japan). We maintained a distance of ~5 cm between the airbrush and the target during matrix coating and allowed sections to dry between coating cycles to minimize delocalization of target compounds.

MALDI-IMS was performed using an Ultra Flextreme MALDI-time-of-flight (TOF) mass spectrometer (Bruker Daltonics, Leipzig, Germany) equipped with an Nd:YAG laser. Accurate MS and MS/MS analyses were performed with a prototype “Mass microscope” (Shimadzu Corporation, Kyoto, Japan). For both instruments, the laser power was optimized to minimize in-source decay of phosphate nucleotides. Data were acquired in the negative reflectron mode with raster scanning using a pitch distance of 100 μm. Each mass spectrum was the result of 300 laser shots at each data point. Signals between *m/z* 50 and 1000 were collected. Image reconstruction was performed using FlexImaging 4.0 software (Bruker Daltonics). Peaks of specific metabolite molecules were assigned by accurate MS analyses with an ion trap TOF instrument (see Supplementary Table S2) as well as by MS/MS on tissues (see Supplementary Figs S7, S8, and S9) according to experimental and presentation guidelines for MALDI-IMS^28^. Optical images of tissue sections were obtained by light microscopy, followed by MALDI-TOF MS imaging of the same section.

### Compound identification for MALDI-IMS

To identify compounds for MALDI-TOF MS imaging, we used one of the following procedures: i) MS/MS on-tissue, ii) a comparison of additional measurements made by CE/ESI/MS on adjacent sections, or iii) a comparison of observed and theoretical *m/z* values^28^. The theoretical and observed *m/z* values can be found in Supplementary Table S2. For more detail, see the supplementary information.

### MALDI imaging of metabolites in tissue sections normalized by CE-MS based quantitative data

To construct apparent content maps for a specific metabolite, we modified a previously reported method^9^. Briefly, an apparent content of a specific metabolite at the i^th^ spot of tissue (*C_i_*) was estimated as follows:


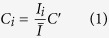


where *C*′ denotes the mean value of a metabolite content determined using CE/ESI/MS in corresponding tissue block, *I_i_* is the maximum intensity of the mass spectra within a specified range at the i^th^ spot, and 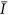
is the median of the maximum intensities of the metabolite from all the spots.

### Statistical analysis

Measurements are reported as mean ± SEM. For single comparisons, we performed an unpaired two-tailed Student’s t-test; for multiple comparisons, we used an analysis of variance (ANOVA) followed by Tukey’s correction for *post hoc* comparisons. Significance was considered at P < 0.05. Statistical analyses were performed using SPSS® software (SPSS Inc., Chicago, IL, USA).

### Animal welfare

According to the American Veterinary Medical Association Recommendations (AVMA Guidelines for the Euthanasia of Animals: 2013 Edition), high-energy microwave irradiation is a humane method for euthanizing small laboratory rodents where unconsciousness is achieved in less than 100 ms with a complete loss of brain function in less than 1 s. During mouse heart fixation, heartbeat completely ceased within 1 s. Therefore, we consider FMW-fixation of the heart to be humane and satisfies the criteria provided by an ethical review at each research institute.

## Additional Information

**How to cite this article**: Sugiura, Y. *et al*. Visualization of *in vivo* metabolic flows reveals accelerated utilization of glucose and lactate in penumbra of ischemic heart. *Sci. Rep*. **6**, 32361; doi: 10.1038/srep32361 (2016).

## Supplementary Material

Supplementary Information
