# Supplementary material for: Visualization of in vivo metabolic flows reveals accelerated utilization of glucose and lactate in penumbra of ischemic heart
Source: Sci Rep. 2016 Sep 1;6:32361. doi: 10.1038/srep32361 (PMC5007669; doi:10.1038/srep32361)
Supplement: Supplementary Information [file srep32361-s1.pdf]

Supplementary Information for

Visualization of *in vivo* metabolic flows reveals accelerated utilization of glucose and lactate in penumbra of ischemic heart

Authors: [1,4]Yuki Sugiura, [2]Yoshinori Katsumata (these authors contributed equally), [2,4]Motoaki Sano*, [1]Kurara Honda, [1,3]Mayumi Kajimura, [2]Keiichi Fukuda, and [1,3]Makoto Suematsu*

Affiliations:

[1]Department of Biochemistry, [2]Department of Cardiology, Keio University School of Medicine, Tokyo, 160-8582, Japan

[3]Exploratory Research for Advanced Technology (ERATO), Japan Science and Technology, Suematsu Gas Biology Project, Tokyo, 102-0076, Japan

[4]Precursory Research for Embryonic Science and Technology (PRESTO), Japan Science and Technology Agency, Tokyo, 102-0076, Japan

*Corresponding author (E-mail address: msano@a8.keio.jp, gasbiology@z6.keio.jp)

Phone: 81-3-5363-3753; FAX: 81-3-5363-3466

Supplementary Methods

Chemicals

$^{13}C_6$-glucose and $^{13}C_3$-lactate were obtained from ISOTEC (Sigma-Aldrich). N-(1-Naphthyl) ethylenediamine dihydrochloride and 9-aminoacridine were obtained from Merck Millipore. All other chemicals were obtained from either Sigma-Aldrich or Wako. All chemicals were HPLC reagent grade.

Mouse model of myocardial ischemia

All animal procedures were conducted in accordance with the Animal Experimentation Guidelines of Keio University School of Medicine and were approved by the Laboratory Animal Care and Use Committee of Keio University [Permission number; 11050-(0), 12094-(1)]. Eight-week-old male C57BL/6J mice (22–26 g; Clea Japan, Tokyo, Japan) were used. They were fed with laboratory chow and allowed free access to water.

Myocardial ischemia was induced by ligating the left anterior descending artery (LAD) as described previously [1]. Briefly, mice were anesthetized with isoflurane (5% for induction, 1.5-2% for maintenance), tracheotomized and mechanically ventilated using a small-animal ventilator (MiniVent, Type 845, Harvard Apparatus, MA, USA). The chest cavity was opened via left thoracotomy to expose the heart such that the LAD coronary artery could be visualized by microscopy. The coronary artery was ligated with a 7-0 silk suture at the site of its emergence from the left atrium. Complete occlusion of the vessel was confirmed by the presence of myocardial blanching in the perfusion bed. Sham-operated animals underwent the same procedure without coronary artery ligation.

Administration of ^{13}C-labelled metabolites

For experiments tracing the metabolic fate of glucose, mice received $^{13}C_6$-glucose (1 mg/g body weight, in saline) by intraperitoneal administration 10 min before LAD ligation,

because at this time point, blood glucose concentration is high enough to trace metabolic pathways of *in vivo* organs by MS [2]. $^{13}C_3$-lactate (27 μg/g body weight, in saline) was administered by retro-orbital injection 1 min before LAD ligation [3]. We have confirmed the elevation of blood lactate concentration to 7-8 mM at this time point, which is within the physiological range (Supplementary Fig. S5 online) [4].

Fixation of heart metabolites by FMW

We used a laboratory microwave instrument (MMW-05 Muromachi Kikai, Tokyo) designed for the euthanasia of laboratory mice and rats (Supplementary Fig. S1 online). This instrument differs from kitchen units, particularly in maximal power output (5 kW) and in having a tightly focused microwave beam. All units direct their microwave energy to a specific anatomical location on the animal. Mice were anesthetized with isoflurane, and placed into a transparent water-jacket holder (Muromachi-Kikai, MH28-HZ). The cone-parts of the holder were filled with ~1 mL of water to help elevate the temperature of the heart as uniformly as possible using microwave energy. Care was taken not to introduce air bubbles. This was then inserted into the instrument in a position such that microwave irradiation was targeted on both brain and heart. For reliable fixation it is important to maintain the animal in the correct position; i.e., the mouse should be straight with its nose touching the top of the cone. The holder is set at the position shown in Supplementary Fig. S1, panel-D; the back end of the holder was set at 43 mm from the entrance of the insertion slot. We found this condition optimal for B6/J mice (8 week old males). Microwave irradiation at 5 kW for 0.96 s elevated the temperature of the heart to above 80°C which is sufficient to inactivate metabolic enzymes, such as acetylcholine esterase [5].

To evaluate the effectiveness of the FMW fixation method, we compared it to two other procedures, i) *rapid-freezing,* in which hearts were isolated immediately after thoracotomy, then frozen in liquid N_2 (total procedure takes ~20 s); ii) *delayed freezing,* in which hearts were isolated 10 min after cervical dislocation to allow postmortem degradation.

Preparation of tissue sections for metabolome and MALDI-imaging analyses

After FMW, hearts were dissected with a surgical knife at room temperature, embedded into a super cryo-embedding medium (SCEM, Section Lab Co. Ltd, Hiroshima, Japan), frozen in liquid N_2, and stored at -80°C. We prepared five sets of short-axis (transverse) tissue sections where each set consisted of a thick 450 µm "block" for quantification of metabolites and an adjacent 8 µm thin "section" for MALDI imaging analyses (see Supplementary Fig. S6 online). The apical two thirds of the left ventricle in sham-operated hearts was subdivided into four short-axis blocks.

The thin sections were cut with a cryomicrotome (CM3050, Leica Microsystems) and thaw-mounted on an indium thin oxide-coated glass slide (BrukerDaltonics, Germany) at -16˚C. Heart tissues subjected to FMW tended to be more fragile than those treated by other methods, often making tissue sectioning challenging. However, embedding the tissue with SCEM medium, which did not interfere with the ionization efficiency of metabolites, helped achieve successful sectioning.

Capillary electrophoresis-electrospray ionization (CE-ESI)-MS

Quantitative metabolome analysis was performed using CE-MS [6]. Briefly, to extract metabolites from the tissue, the frozen tissue block embedded in SCEM medium together with internal control compounds (see below) was homogenized in ice-cold methanol (500 µL) using a manual homogenizer (Finger Masher (AM79330); Sarstedt, Tokyo, Japan), followed by the addition of an equal volume of chloroform and 0.4 times the volume of ultrapure water (LC/MS grade; Wako). The suspension was then centrifuged at 15,000 *g* for 15 min at 4°C. After centrifugation, the aqueous phase was ultra-filtered using an ultrafiltration tube (Ultrafree-MC, UFC3 LCC NB; Human Metabolome Technologies, Tsuruoka, Japan). The filtrate was concentrated with a vacuum concentrator (SpeedVac; Thermo, Yokohama, Japan); this condensation process helps quantitate trace levels of metabolites. The concentrated filtrate was dissolved in 50 µL of ultrapure water and used for CE-MS.

All CE-MS experiments were performed using an Agilent CE System equipped with an air pressure pump, an Agilent 6520 Accurate Q-Tof mass spectrometer, an Agilent 1200 series isocratic high-performance LC pump, 7100 CE-system, a G1603A Agilent CE-MS adapter kit, and a G1607A Agilent CE-MS sprayer kit (Agilent Technologies).

Isomeric species, such as glucose 1-phosphate, glucose 6-phosphate, and fructose 6-phosphate, can be hard to distinguish by MS/MS *per se*. However, we took advantage of the CE/ESI/MS system [6], in which these species are eluted at a different retention time by capillary electrophoresis due to their differential mobility and/or chemical properties. The system separates isobaric or isomeric compounds effectively.

Electrospray interface and MS conditions

ESI–MS was conducted in negative ion mode, and the capillary voltage was set at 3.5 kV. The fragmenter, skimmer, and Oct RFV voltage were set to 97, 62, and 550 V, respectively. The nitrogen nebulizer pressure was set at 10 psi, and a flow rate of drying nitrogen gas (heater temperature 240°C) was maintained at 4 L/min. Automatic recalibration of each acquired spectrum was performed using reference masses of reference standards; namely, TFA anion (m/z 112.985587) and HP-0921 compound anion (m/z 1033.988109) (G1969-8500, API-TOF Reference Mass Solution Kit, Agilent Technologies). We confirmed that the mass error was within 10 ppm for the targeted metabolites. Exact mass data were acquired at a rate of 1.4 spectra/s over a 61−1050 m/z range (0.713 s duty cycle).

Quantification of metabolites by internal and external standards

We used both internal (added to the tissue before extraction) and external (used to produce calibration curves for each compound) standard compounds for concentration calculation. The detailed method is as follows:

Internal standard (IS) compounds

We used 2-morpholinoethanesulfonic acid (MES) and 1,3,5-benzenetricarboxylic acid (trimesate) as ISs for anionic metabolites. These compounds are not present in the tissues; thus, they serve as ideal standards. Loss of endogenous metabolites during sample preparation was corrected by calculating the recovery rate (%) for each sample measurement.

External standard (ES) compounds

An external calibration curve was used to calculate the absolute abundance of metabolites. Before sample measurement, we measured the mixture of authentic compounds of target metabolites at three different concentrations (50, 10 and 5 µM for adenine nucleotides, and 500, 100 and 50 µM for lactate) in ultrapure water to generate calibration curves. Quantification (amount of metabolites, nmol/mg tissue or nmol/mg protein) was performed by comparing the IS-normalized peak areas against the calibration curves.

Liquid chromatography-tandem mass spectrometry

The amount of non-labeled and $^{13}C_6$-glucose in the heart was quantified using liquid chromatography-tandem mass spectrometry (LC-MS/MS). Briefly, a triple-quadrupole mass spectrometer equipped with an electrospray ionization (ESI) ion source (LCMS-8030; Shimadzu Corporation, Kyoto, Kyoto, Japan) was used in the negative-ESI and multiple reaction monitoring (MRM) modes. The samples were resolved on the PC-HILIC S3 column (150 × 2.0 mm, i.d., 4 µm particle), and separated using mobile phase A (water) and mobile phase B (acetonitrile) at a flow rate of 0.8 mL/min and a column temperature of 40°C. Non-labeled- and $^{13}C_6$-labeled-glucose were quantified by ion transitions from *m/z* 179 to *m/z* 89, and *m/z* 185 to *m/z* 92 respectively.

Matrix coating and MALDI-IMS acquisition

Prior to matrix coating, the tissue slices were placed in desiccant for 10 min and allowed to equilibrate to room temperature. We used 9-aminoacridine as a matrix (10 mg/mL, dissolved

in 80% ethanol) and manually spray-coated tissues sections with the solution using an artistic air-brush (Procon Boy FWA Platinum 0.2-mm caliber airbrush, Mr. Hobby, Tokyo, Japan). We maintained a distance of ~5 cm between the air-brush and the target during matrix coating, and allowed sections to dry between coating cycles to minimize delocalization of target compounds.

MALDI-IMS was performed using an Ultra Flextreme MALDI-time-of-flight (TOF) mass spectrometer (Bruker Daltonics, Leipzig, Germany) equipped an Nd:YAG laser. Accurate MS and MS/MS analyses were performed with a prototype “Mass microscope” (Shimadzu Corporation, Kyoto, Japan). For both instruments, the laser power was optimized to minimize in-source decay of phosphate nucleotides. Data were acquired in the negative reflectron mode with raster scanning using a pitch distance of 100 μm. Each mass spectrum was the result of 300 laser shots at each data point. Signals between *m/z* 50 and 1000 were collected. Image reconstruction was performed using FlexImaging 4.0 software (Bruker Daltonics). Peaks of specific metabolite molecules were assigned by accurate MS analyses with an ion trap TOF instrument (see Supplementary Table S2 online) as well as by MS/MS on tissues (see Supplementary Fig. S7, S8, and S9 online) according to experimental and presentation guidelines for MALDI-IMS [7]. Optical images of tissue sections were obtained by light-microscopy, followed by MALDI-TOF MS imaging of the same section.

Compound identification for MALDI-IMS:

To identify compounds for MALDI-TOF MS imaging, we used one of the following procedures: i) MS/MS on-tissue, ii) a comparison of additional measurements made by CE/ESI/MS on adjacent sections, or iii) a comparison of observed and theoretical *m/z* values [7]. The theoretical and observed *m/z* values can be found in Supplementary Table 2.

It is not possible to separate $^{13}C_2$-glu and $^{13}C_3$-gln by MALDI-imaging. To solve this problem, we utilized CE/ESI/MS analyses to determine $^{13}C_2$-glu and $^{13}C_3$-gln content in adjacent sections from the same sample. By doing so, we confirmed that the level of $^{13}C_3$-gln was below the level of detection. This led us to conclude that the *m/z* value, 149.13, in

negative ion mode represents $^{13}C_2$-glu and that the sample is less likely to be contaminated with $^{13}C_3$-gln.

MALDI imaging of metabolites in tissue sections normalized by CE-MS based quantitative data

To construct apparent content maps for a specific metabolite, we modified a previously reported method [8]. Briefly, an apparent content of a specific metabolite at the ith spot of tissue (C_i) was estimated as follows:

$$C_i = \frac{I_i}{\bar{I}} C'$$

where C' denotes the mean value of a metabolite content determined using CE/ESI/MS in corresponding tissue block, I_i is the maximum intensity of the mass spectra within a specified range at the ith spot, and $\bar{I}$ is the median of the maximum intensities of the metabolite from all the spots.

Statistical analysis

Measurements are reported as mean ± SEM. For single comparisons, we performed an unpaired two-tailed Student’s t-test; for multiple comparisons, we used an analysis of variance (ANOVA) followed by Tukey’s correction for *post hoc* comparisons. Significance was considered at $P < 0.05$. Statistical analyses were performed using SPSS® software (SPSS Inc., Chicago, IL, USA).

Animal welfare

According to the American Veterinary Medical Association Recommendations (AVMA Guidelines for the Euthanasia of Animals: 2013 Edition), high-energy microwave irradiation is a humane method for euthanizing small laboratory rodents where unconsciousness is achieved in less than 100 ms with a complete loss of brain function in less than 1 sec.

During mouse heart fixation, heartbeat completely ceased within 1 sec. Therefore, we consider FMW-fixation of the heart to be humane and satisfies the criteria provided by an ethical review at each research institute.

Reference

1 Anzai, A. et al. Regulatory role of dendritic cells in postinfarction healing and left ventricular remodeling. Circulation **125**, 1234-1245 (2012).

2 Sugiura, Y., Honda, K., Kajimura, M. & Suematsu, M. Visualization and quantification of cerebral metabolic fluxes of glucose in awake mice. Proteomics **14,** 829-838 (2014).

3 Yardeni, T., Eckhaus, M., Morris, H. D., Huizing, M. & Hoogstraten-Miller, S. Retro-orbital injections in mice. Lab anim. **40,** 155-160 (2011).

4 Suhara, T. et al. Inhibition of the oxygen sensor PHD2 in the liver improves survival in lactic acidosis by activating the Cori cycle. Proc. Natl. Acad. Sci. USA **112**, 11642-11647 (2015).

5 Moroji, T., Takahashi, K., Ogura, K., Toishi, T. & Arai, S. Rapid microwave fixation of rat brain. J. Microw. Power **12,** 273-286 (1977).

6 Soga, T. et al. Quantitative metabolome analysis using capillary electrophoresis mass spectrometry. J. Proteome Res. **2,** 488-494 (2003).

7 McDonnell, L. A. et al. Discussion point: reporting guidelines for mass spectrometry imaging. Anal. Bioanal. Chem. **407,** 2035-2045 (2015).

8 Hattori, K. et al. Paradoxical ATP elevation in ischemic penumbra revealed by quantitative imaging mass spectrometry. Antioxid. Redox Signal. **13,** 1157-1167 (2010).

Supplementary Figures

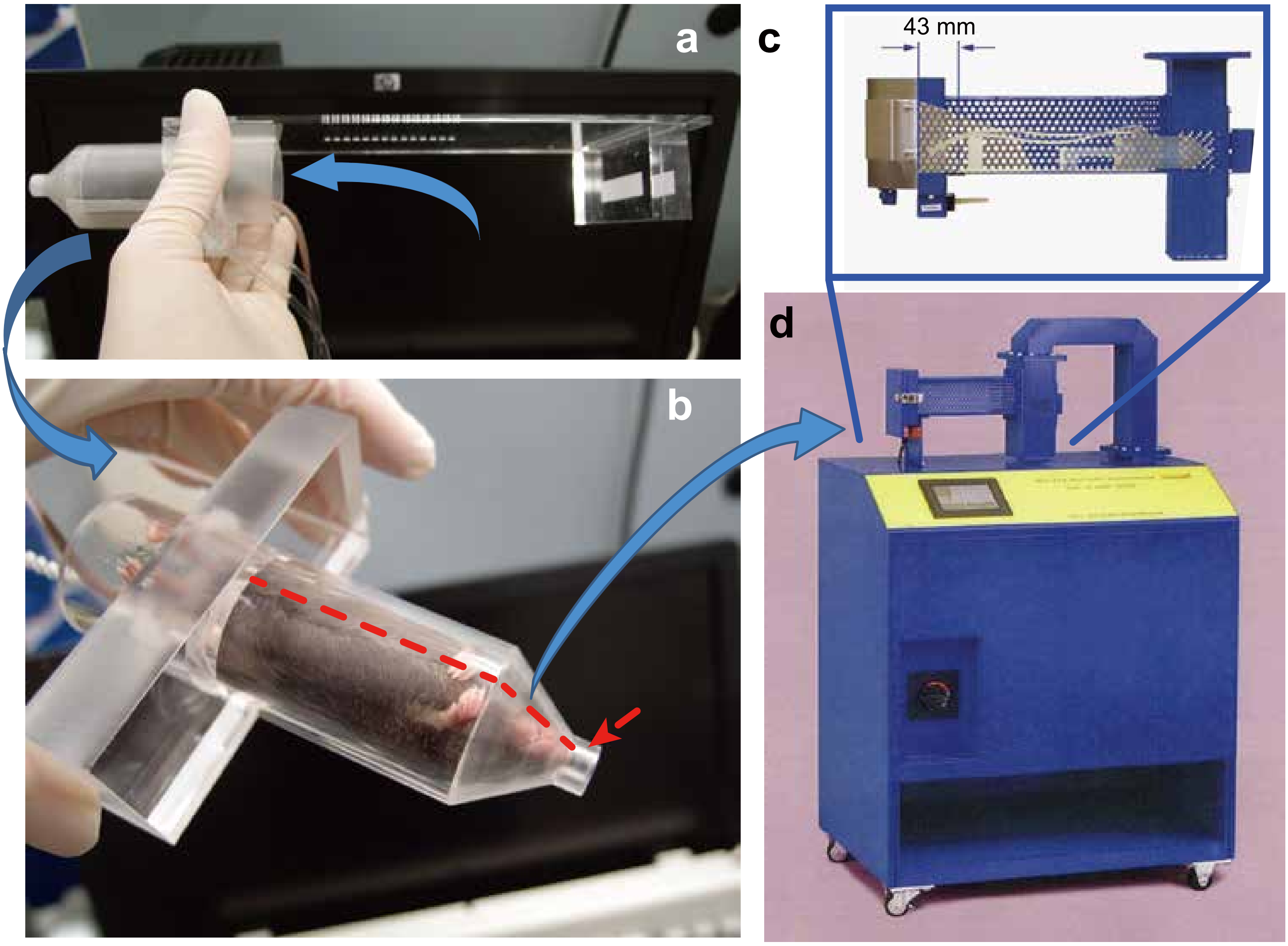

Figure S1. Protocol for snap-fixation of animal heart metabolism by focused microwave irradiation (FMW) treatment

Mice were subjected to FMW with a purpose-built instrument (MMW-05, Muromachi Kikai, Tokyo) (d), using the following parameters: power 5.5 kW, irradiation time 0.94 sec.

(a-b) Placement of the anesthetized animal in the restrainer.

For reliable fixation, correct animal placement inside the restrainer is very important. The posture of the mouse inside the restrainer should also be precise. For this to be achieved: (1) Reverse the restrainer (a) and position the anesthetized mouse in an erect posture so as to touch its nose to the top of the cone (b). (2) Quickly reverse the restrainer and position a mouse body stopper.

(c-d) Exposure conditions for the mouse heart.

It is also important to set the restrainer at an optimal position within the microwave instrument. If the restrainer is positioned suboptimally, it will be difficult to achieve uniform heating. The positioning of the restrainer is critical for reproducibility. For a typical B6/J mice (8 weeks aged male), uniform heating can be obtained with the mouse holder at the position shown in panel-D; the back end of the folder is set at 43 mm from the entrance of the insertion slot.

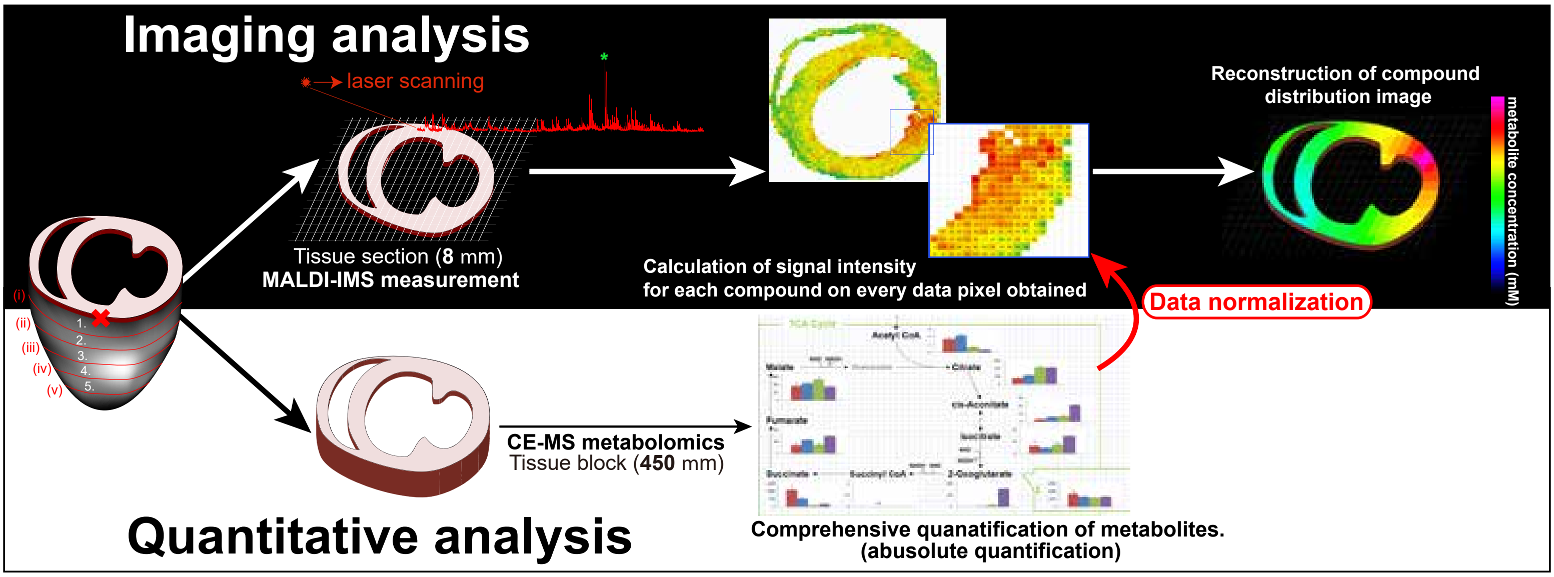

Figure S2. Quantitative imaging of cardiac metabolites by Q-IMS

Application of matrix-assisted laser desorption/ionization imaging mass spectrometry (MALDI-IMS), combined with focused microwave irradiation (FMW) to rapidly fix tissue metabolism, accurately and reproducibly visualized regional contents of *in vivo* endogenous metabolites and spatial differences of metabolites in mice. MALDI-IMS reconstructed by capillary electrophoresis–mass spectrometry (CE-MS)-based data could evaluate the fluctuation of metabolites between different mouse tissue slices more quantitatively.

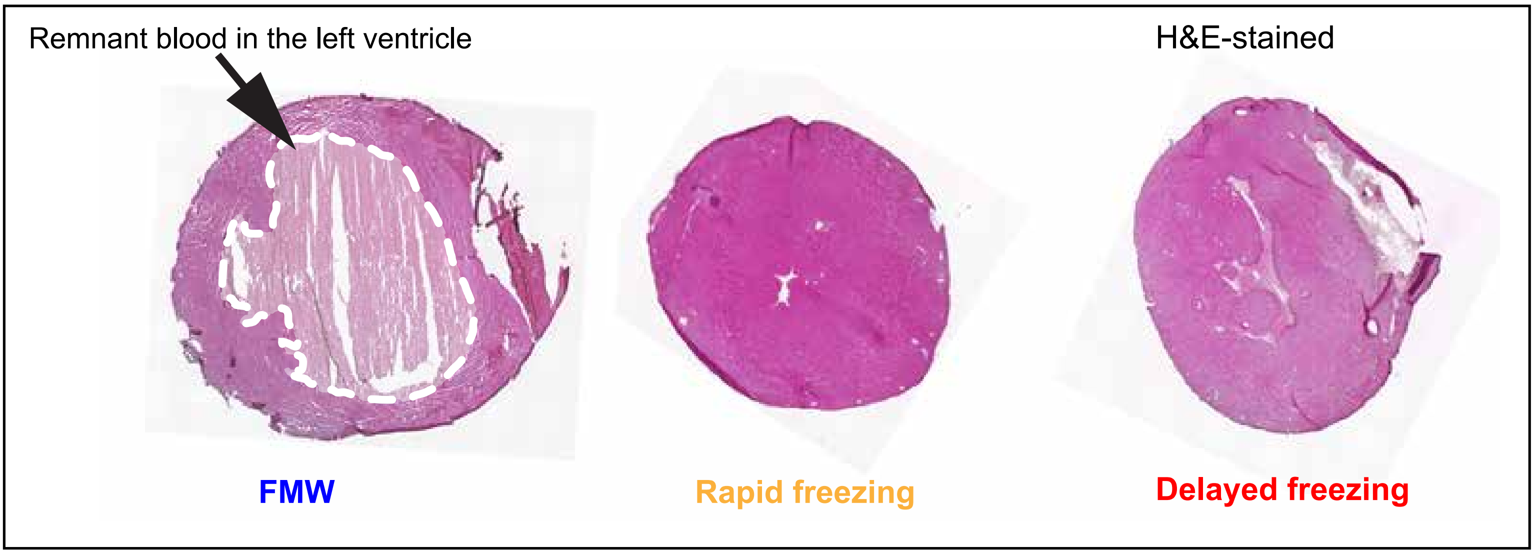

Figure S3. Sections of heart tissues

Sections of heart tissue stained with hematoxylin and eosin following focused microwave irradiation at 5 kW for 0.94 sec, rapid-freezing and delayed-freezing methods.

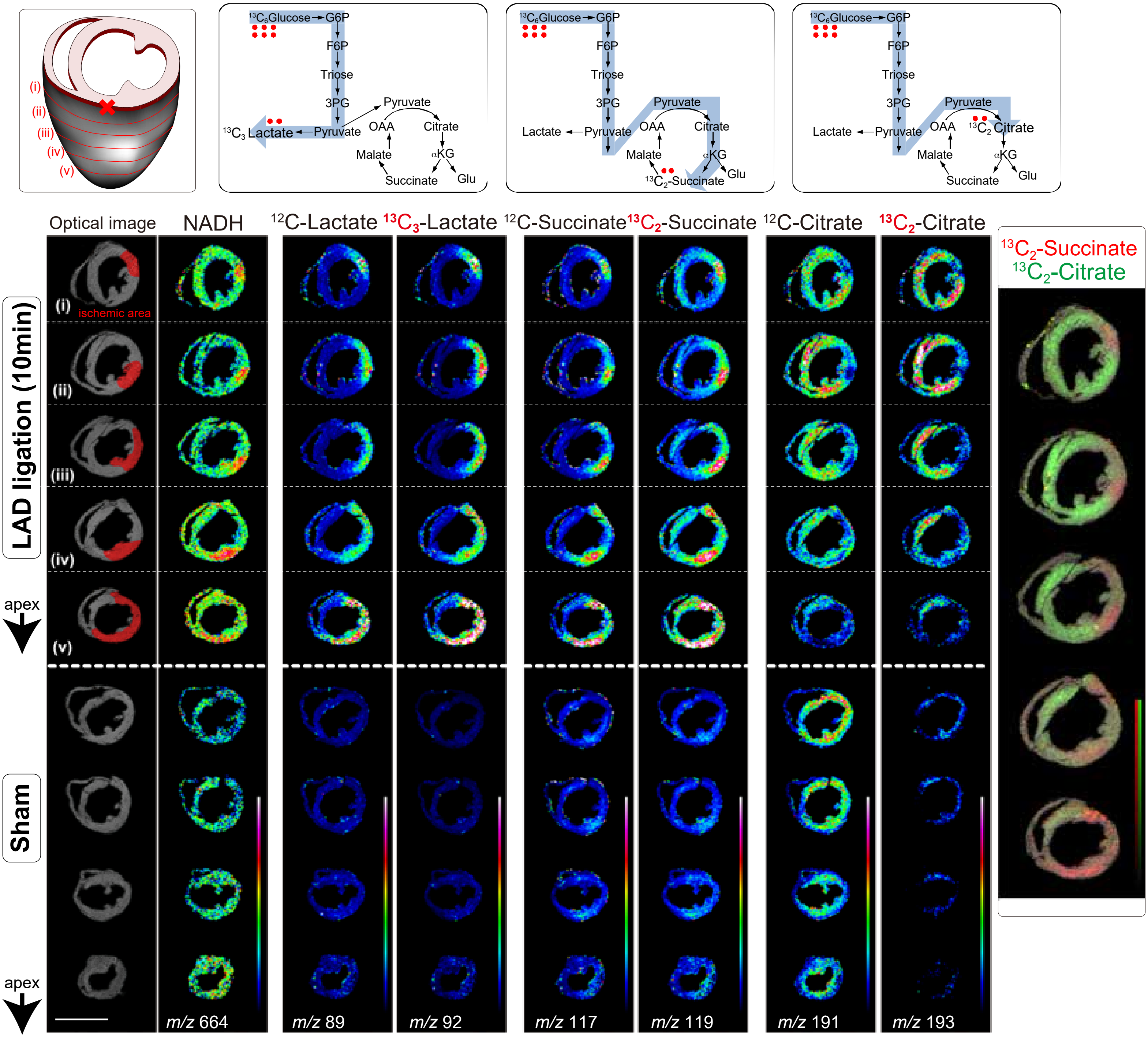

Figure S4. Visualization of the $^{13}C_6$ -glucose with a NEDC matrix

Two-dimensional maps of non-labeled metabolites and ^{13}C labelled metabolites in the heart following LAD ligation were reconstructed from matrix-assisted laser desorption/ionization imaging mass spectrometry (MALDI-IMS) combined with N-(1-naphthyl) ethylenediamine dihydrochloride (NEDC) as a matrix, and shown as an overlay on optical heart section images. Images were normalized with total ion current (TIC). The red region in the optimal image indicates the area with increased NADH as a metabolic indicator for the ischemic region. The right lane is the merge of $^{13}C_2$-succinate and $^{13}C_2$-citrate. Experiments were repeated at least three times with reproducible results. Representative images are shown.

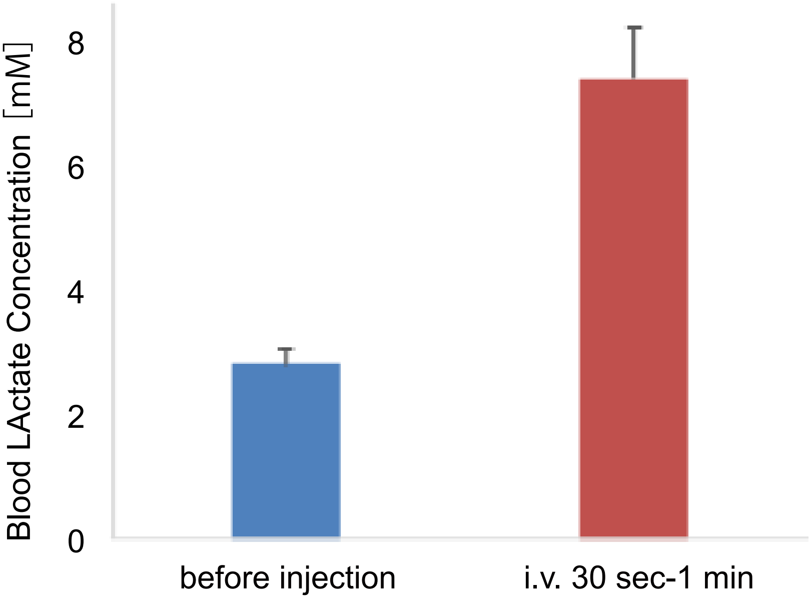

Figure S5: Blood lactate concentration before and after $^{13}C_3$-lactate administrated mice

The blood lactate levels were measured using Lactate Pro2 Test Meter and Lactate Pro Test Strip.

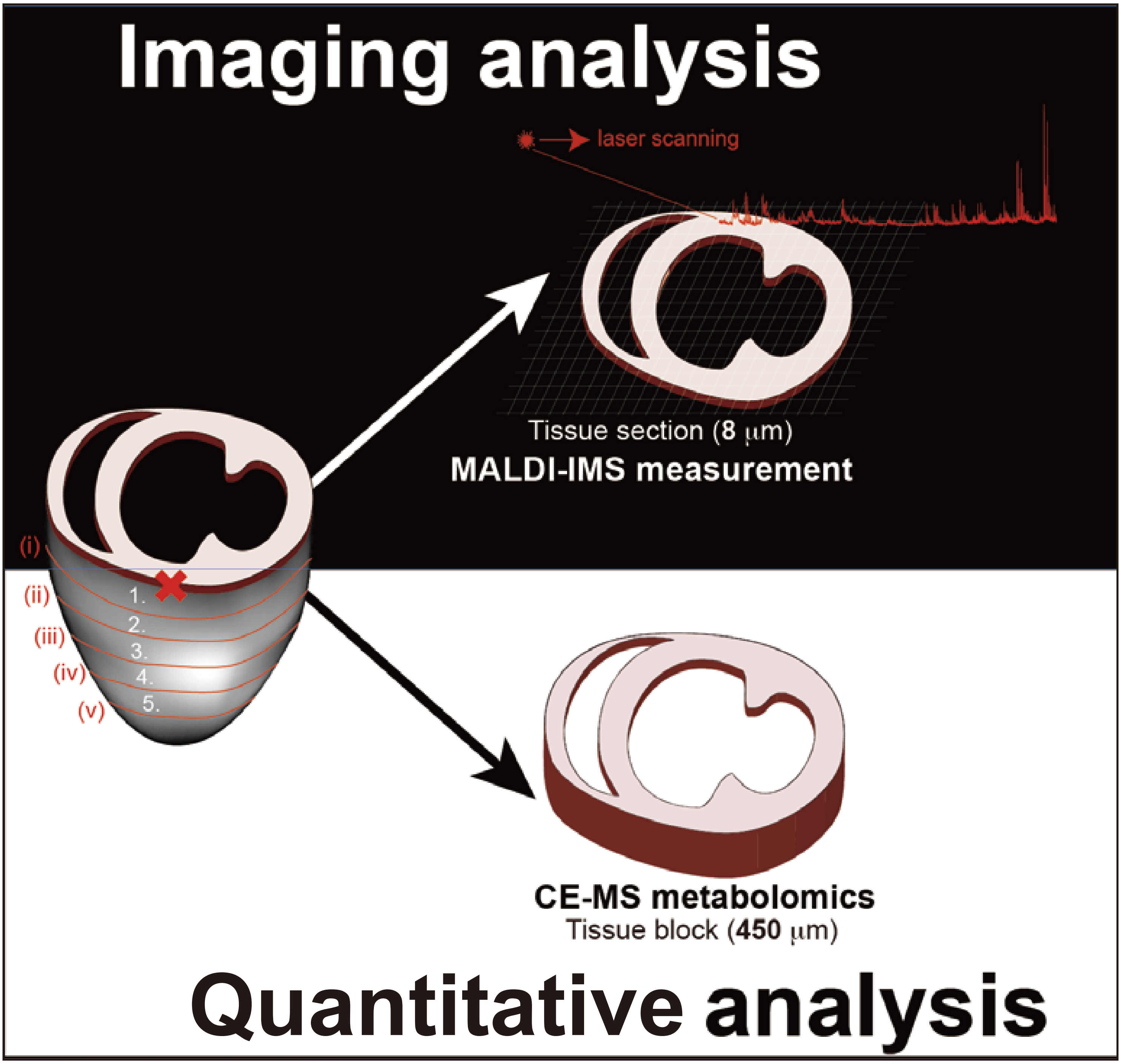

Figure S6. Schematic to highlight designated sampling areas for IMS and CE/ESI/MS analyses

Five transverse blocks of 450-μm thickness covering the point of the ligation to the apex were harvested. From each block, an 8-μm section was cut and thaw-mounted on indium-tin-oxide (ITO) coated glass for IMS.

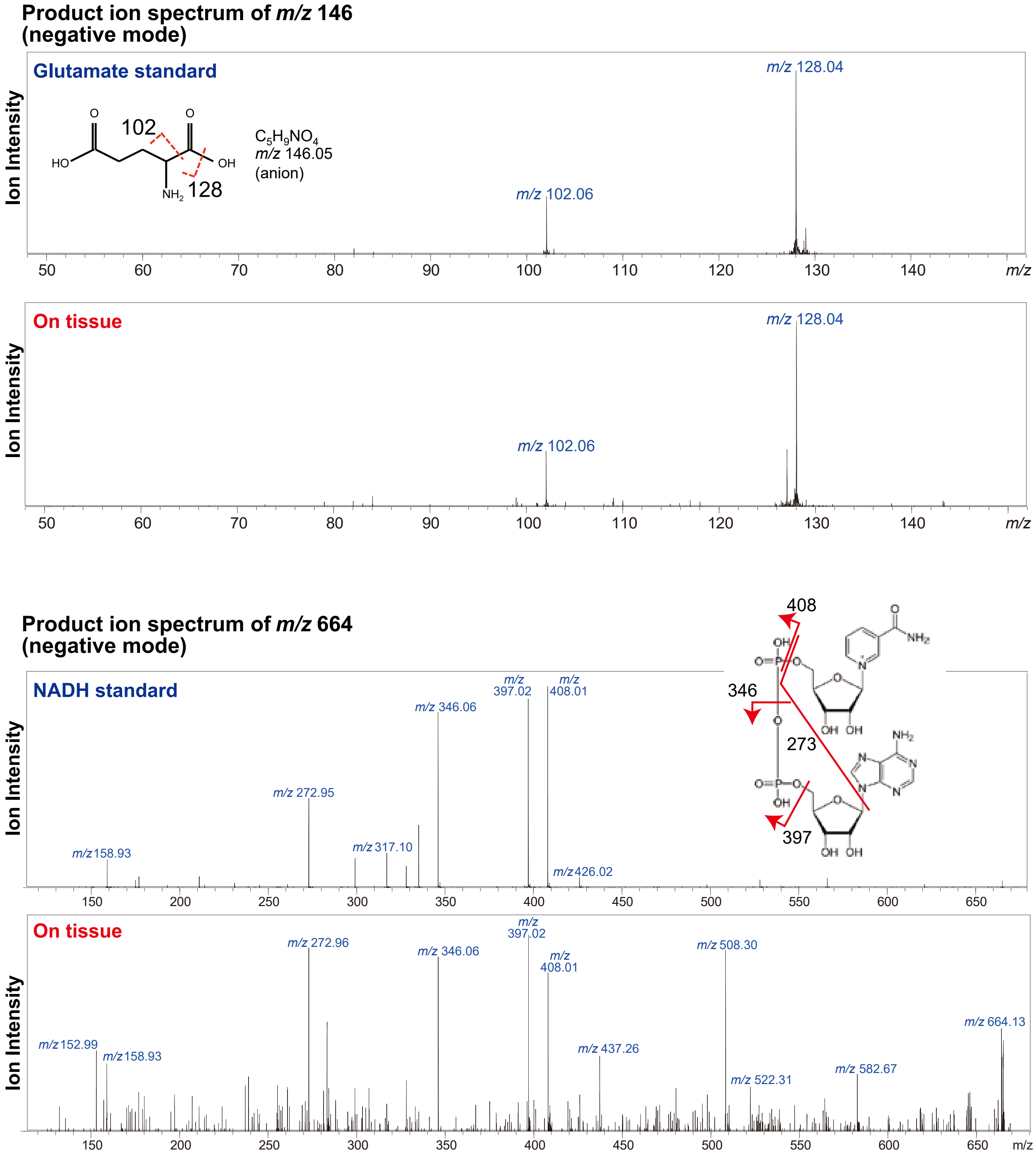

Figure S7. Tandem MS analysis to identify glutamate and NADH

The chemical structures show the assignments of the diagnostic fragments. Comparisons of tissue MS/MS spectra with ion peaks at *m/z* 146 (negative-ion mode) and *m/z* 664 (negative-ion mode) obtained from tissue or glutamate and NADH standards, respectively. The similarity of the two spectra was used to assign the metabolites as glutamate and NADH.

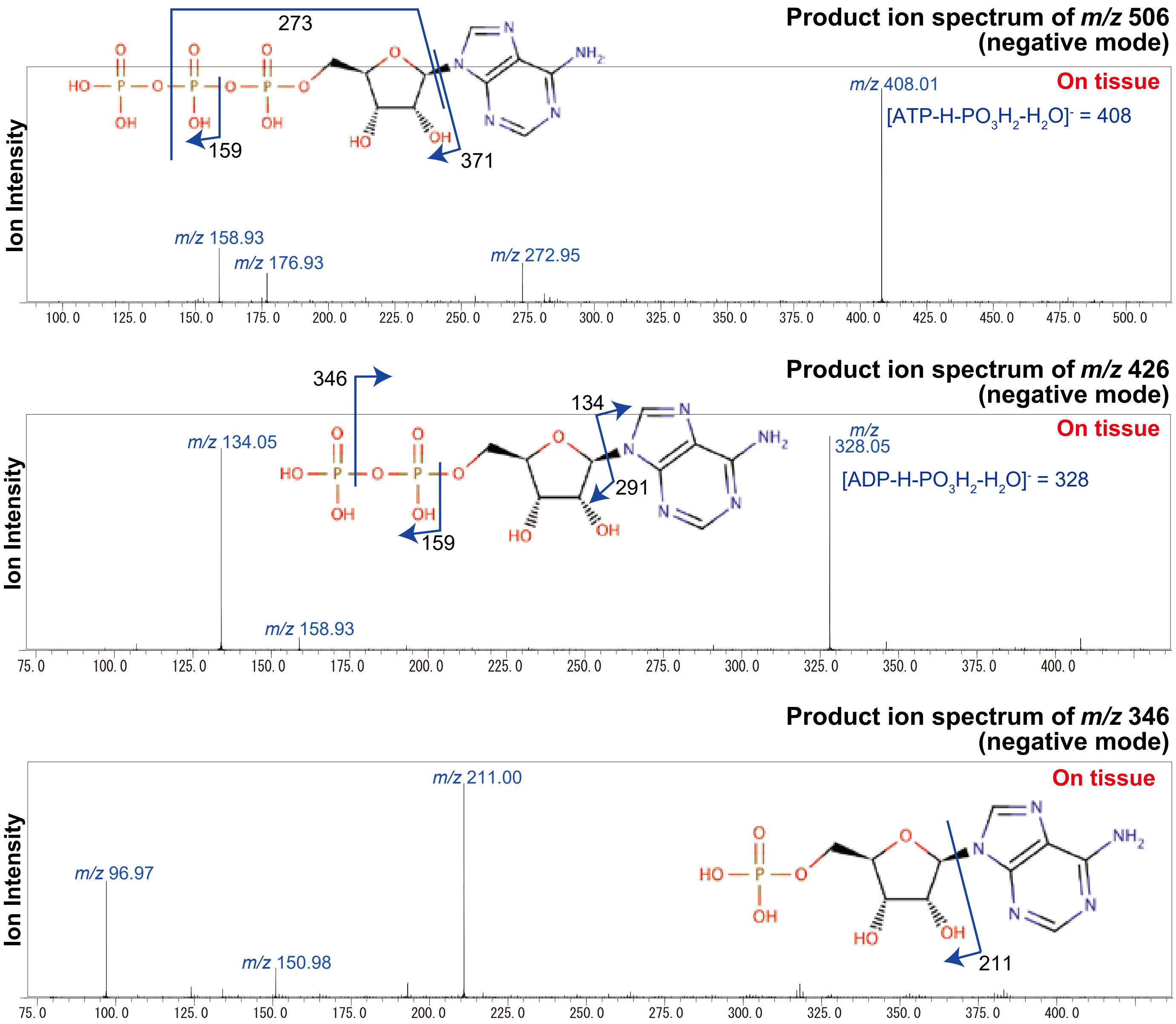

Figure S8. Tandem MS analysis to identify adenine nucleotides

The chemical structures show the assignments of the diagnostic fragments. Comparisons of tissue MS/MS spectra with negatively charged ion peaks at *m/z* 506, *m/z* 426 and *m/z* 346 obtained on tissue. Fragment peaks observed in the product ion spectra were assigned with reference to the METLIN database (https://metlin.scripps.edu/index.php).

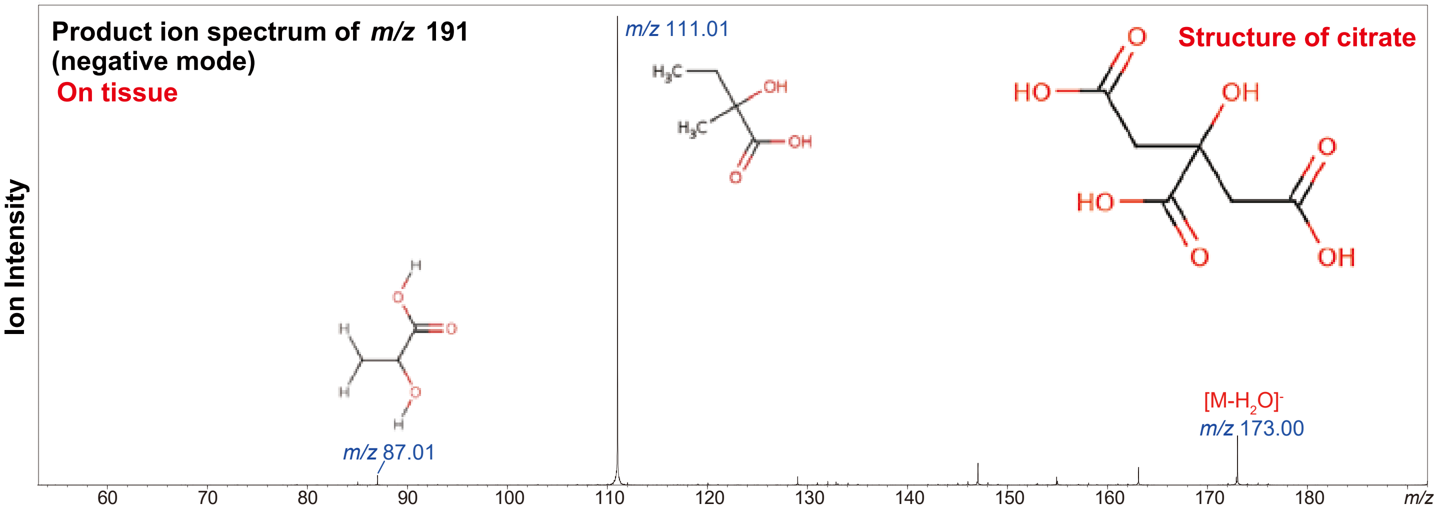

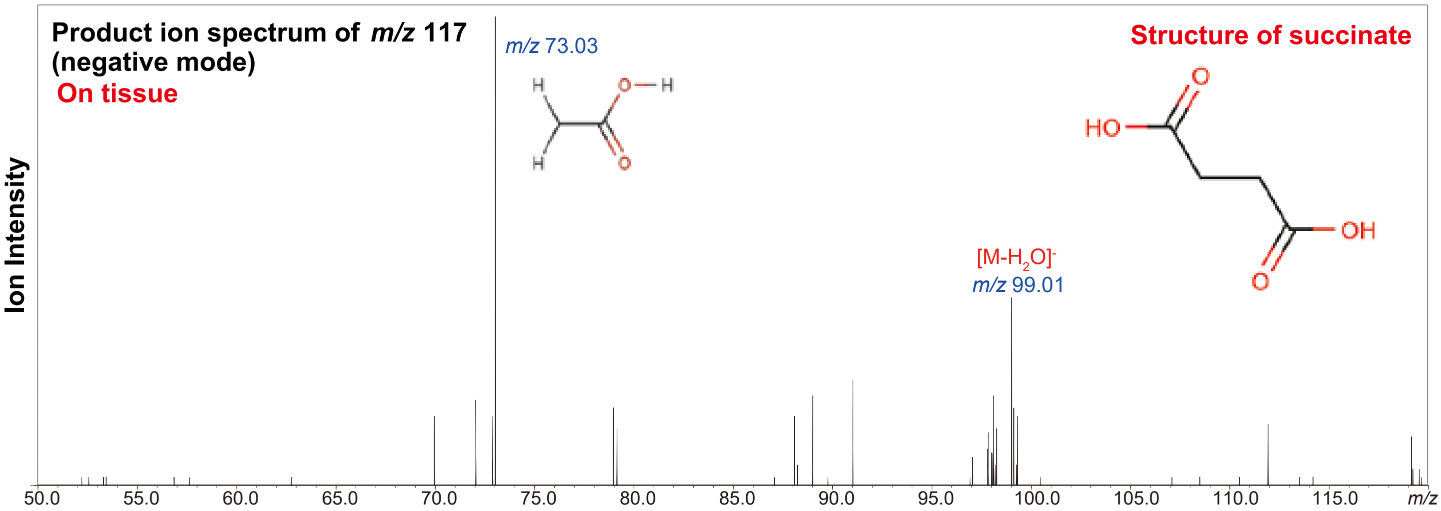

Figure S9. Tandem MS analysis to identify organic acids

The chemical structures show the assignments of the diagnostic fragments. Comparisons of MS/MS spectra with negatively charged ion peaks at *m/z* 191 and *m/z* 117 obtained in tissue. Fragment peaks observed in the product ion spectra were assigned in reference to METLIN data base (https://metlin.scripps.edu/index.php).

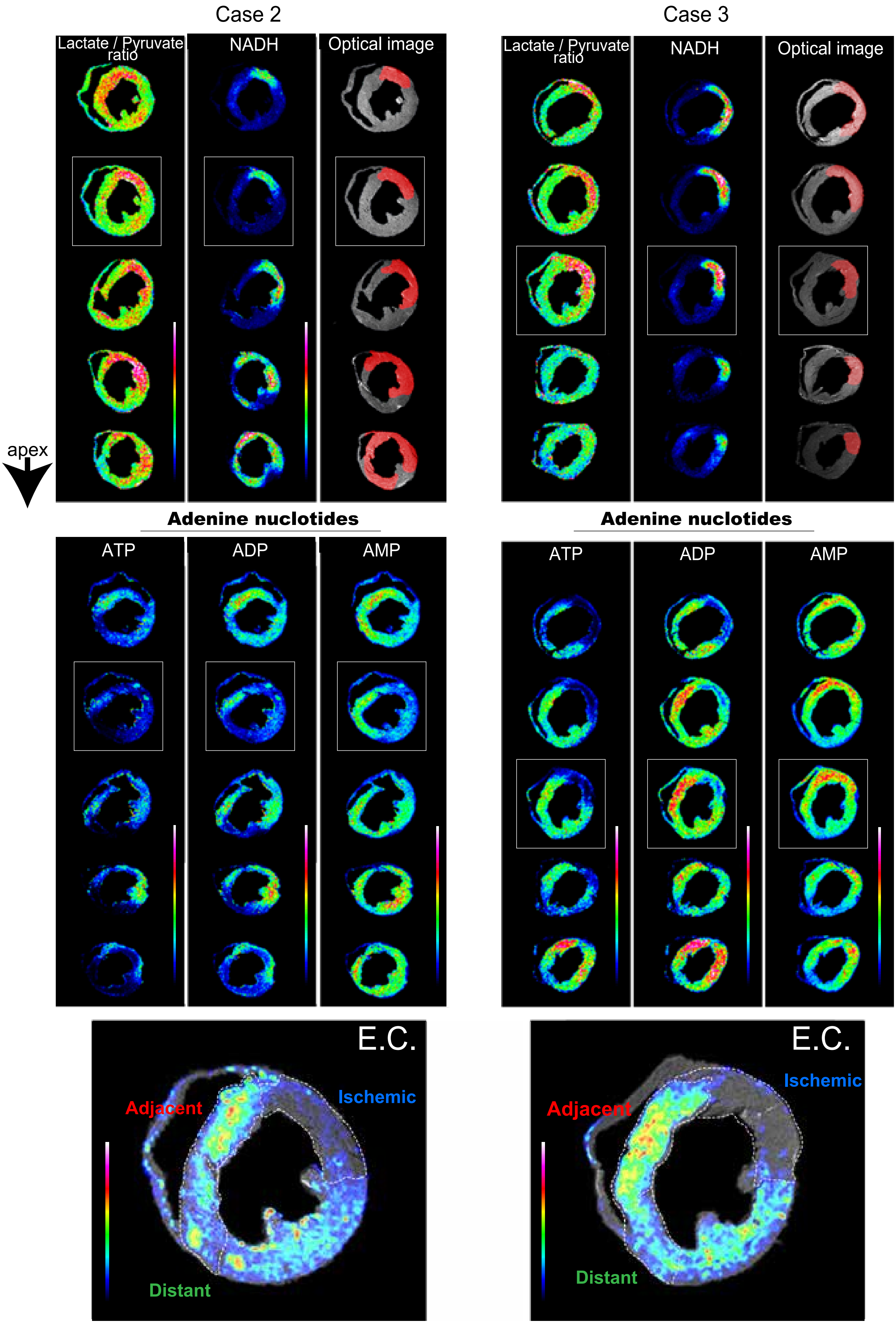

Figure S10. Quantitative imaging of cardiac metabolites

Two-dimensional maps of short-axis sections obtained from LAD-ligated hearts were reconstructed with matrix-assisted laser desorption/ionization imaging mass spectrometry (MALDI-IMS) of metabolites. The bottom lanes show maps of energy charge (EC). Two sets of results obtained from independent experiments are shown. The red region in the optimal image indicates the area with increased NADH as a metabolic indicator for the ischemic region.

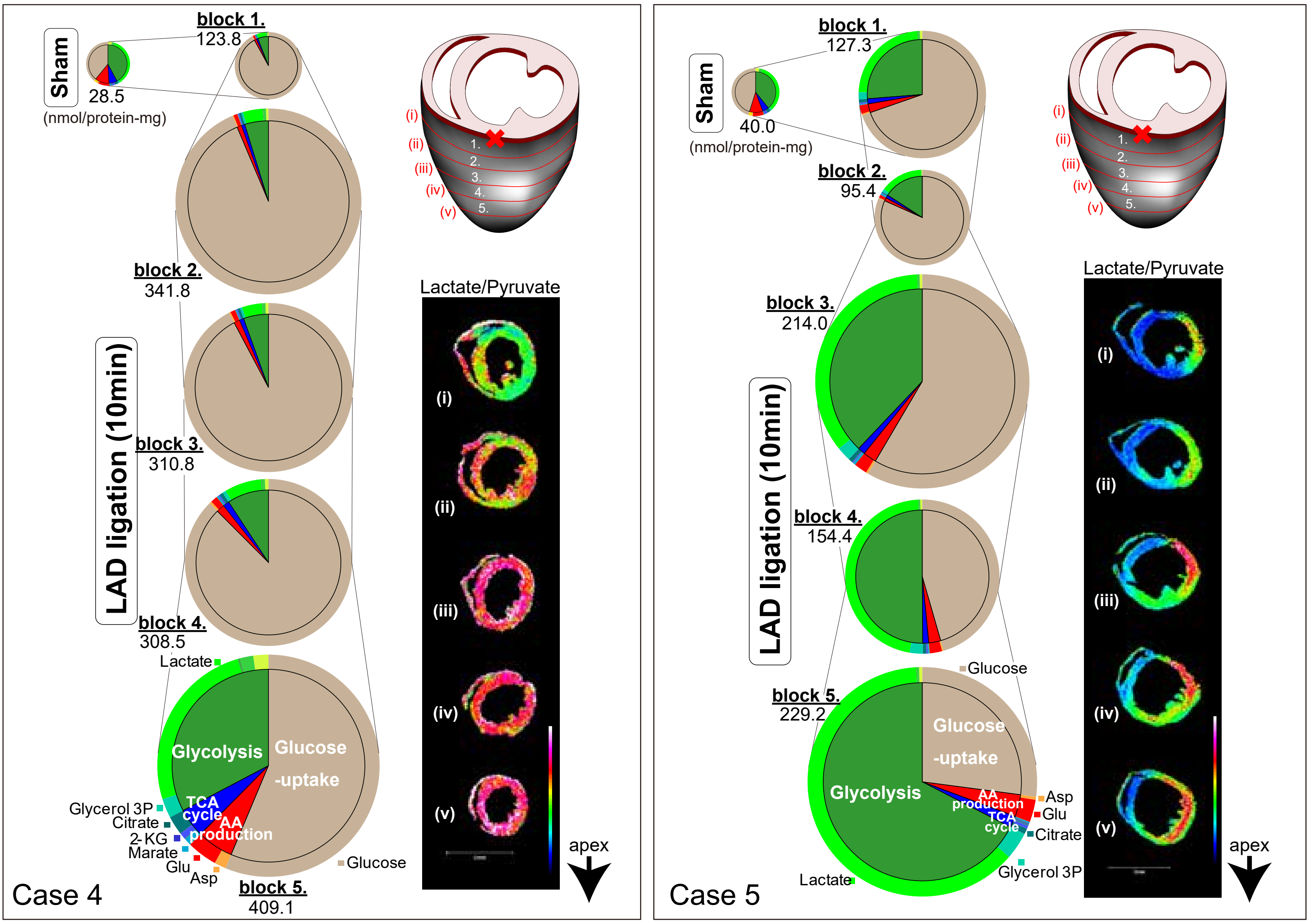

Figure S11. Pathway-tracing analysis using $^{13}C_6$-glucose in the hearts

5 blocks obtained from LAD-ligated hearts were used for capillary electrophoresis–mass spectrometry (CE-MS) quantification of ^{13}C-containing metabolites. Data obtained from different blocks were represented with pie charts. Two sets of results obtained from independent experiments are shown.

Glycolysis	CE/MS	IMS
Glucose 1 -phosphate	○	○
Glucose 6-phosphate	○	×
Fructose 6-phosphate	○	×
Fructose 1,6-diphosphate	○	○
Dihydroxyacetonephosphate	○	×
2,3-Diphosphoglycerate	○	○
3-Phosphoglycerate	○	×
Phosphoenolpyruvate	○	○
Pytuvate	○	○
Lactate	○	○

Glycerol phosphate shuttle	CE/MS	IMS
Glycerol 3-phosphate	○	×

TCA Cycle	CE/MS	IMS
Acetate CoA	○	×
citrate	○	×
cis-Aconitate	○	○
Isocitrate	○	×
2-Oxoglutarate	○	×
Succinyl CoA	○	×
Succinate	○	○
Fumarate	○	○
Malate	○	○

Pentose phosphate pathway	CE/MS	IMS
Glucose 6-phosphate	○	×
6-Phosphogluconate	○	×
Ribulose 5-phosphate	○	×
Ribose 5-phosphate	○	×
D-Sedoheptulose 7-phosphate	○	×
Fructose 6-phosphate	○	○

Purine Pyrimidine metabolism	CE/MS	IMS
Uric acid	○	×
Allantoin	○	×
β-Ala	○	×

Tryptophan metabolism	CE/MS	IMS
Anthranilate	○	×

Amino Acids	CE/MS	IMS
Gly	○	○ *
Ala	○	○ *
Ser	○	○ *
Thr	○	○ *
Val	○	○ *
Ile	○	○ *
Leu	○	○ *
Lys	○	○ *
Arg	○	○ *
His	○	○ *
Tyr	○	○ *
Phe	○	○ *
Trp	○	○ *
Met	○	○ *
Cys	○	○ *
Pro	○	○ *
Gln	○	○ *
Glu	○	○ *
Asn	○	○ *
Asp	○	○ *

Fatty acid metabolism	CE/MS	IMS
L-Carnitine	○	○
CoA	○	×
Acetyl CoA	○	×
Succinyl CoA	○	×
HMG CoA	○	×
Malonyl CoA	○	×
n-Propionyl CoA	○	×

Urea cycle	CE/MS	IMS
glu	○	○ *
Ornithine	○	×
Citrulline	○	○
Arg	○	○ *
Creatine	○	○
Creatine phosphate	○	○
Creatinine	○	×
Hypoxyproline	○	○

Table S1

List of metabolites that can be visualized by capillary electrophoresis–mass spectrometry (CE-MS) or matrix-assisted laser desorption/ionization imaging mass spectrometry (MALDI-IMS). Note that the number of metabolites that can be visualized by IMS is smaller than that measurable by CE-MS.

Nucleic Acids	CE/MS	IMS
Adenine	○	×
Guanine	○	×
Cytosine	○	×
Uracil	○	×
Adenosine	○	○
Guanosine	○	○
Cytidine	○	×
Uridine	○	○
Inosine	○	○
AMP	○	○
GMP	○	○
CMP	○	×
TMP	○	×
IMP	○	○
ADP	○	○
GDP	○	○
CDP	○	×
TDP	○	×
ATP	○	○
GTP	○	○
CTP	○	×
TTP	○	×
UTP	○	○
dATP	○	×
dCTP	○	×
Hypoxanthine	○	×
Xanthiene	○	×
NAD	○	×
NADH	○	○
NADP	○	×
NADPH	○	○
FAD	○	×

Methylated products	CE/MS	IMS
Met	○	×
S-Adenosyl-L-methionine	○	○
Spermidine	○	○
Spermine	○	×
S-Adenosyl-L-homocysteine	○	○
Cystathionine	○	×
Homoserine	○	×
Hypotaurine	○	×
Taurine	○	○
Thiotaurine	○	×
Glutathione, reduced form	○	○
Glutathione, oxidized form	○	○
N-Methyl-Arg	○	×
ADMA	○	×
SDMA	○	×
8-Methyl-cGMP	○	×

Mescle metabolism	CE/MS	IMS
Creatine	○	○
Creatine phophate	○	○
Creatinene	○	×

Nucleic acid synthesis	CE/MS	IMS
5-Phosphoribosyl-pyrophosphate	○	×

Miscellaneous	CE/MS	IMS
Carnosine	○	×
Ophthalmic	○	×
Homolanthionine	○	×

catechlamine metabolism	CE/MS	IMS
Dopamine	○	×
Epinephrine	○	×

Table S1

List of metabolites that can be visualized by capillary electrophoresis–mass spectrometry (CE-MS) or matrix-assisted laser desorption/ionization imaging mass spectrometry (MALDI-IMS). Note that the number of metabolites that can be visualized by IMS is smaller than that measurable by CE-MS.

Name of compounds	*m/z* theoretical	observed	⊿ (Da)	⊿ (ppm)	matrix
Lactate	89.024	89.022	0.002	-22.4658519	9AA, NEDC
Pyruvate	87.009	87.007	0.002	-22.9861279	9AA, NEDC
Succinate	117.0193	117.017	0.0023	-19.6548774	9AA, NEDC
Citrate	191.0197	191.018	0.0017	-8.89960564	NEDC
Glutamate	146.0459	146.045	0.0009	-6.16244619	9AA, NEDC
AMP	346.0558	346.05	0.0058	-16.7603028	9AA
ADP	426.0221	426.015	0.0071	-16.6658021	9AA
ATP	505.9885	505.98	0.0085	-16.7988008	9AA
NADH	664.1175	664.092	0.0255	-38.3968198	9AA

Table S2 Mass accuracy of metabolite ions detected on heart tissue sections.
